# Effects of Orientational and Conformational Ordering on Isotactic Polypropylene Crystallization

**DOI:** 10.1021/acs.macromol.5c03407

**Published:** 2026-01-14

**Authors:** Anderson D. S. Duraes, Wenlin Zhang

**Affiliations:** Department of Chemistry, 3728 Dartmouth College, 41 College Street, Hanover, New Hampshire 03755, United States

## Abstract

We employ atomistic nonequilibrium molecular dynamics (NEMD) simulations to investigate the effects of homogeneous shear and uniaxial extensional flows on the crystallization of isotactic polypropylene (iPP) oligomers. Extensional flows induce stronger, molecular-weight-dependent alignment in iPP, whereas chains in shear flows exhibit weaker alignment that is independent of chain length. While both flow types promote uniaxial alignment of polymer segments, neither induces helical conformational order in iPP. Upon quenching below the melting temperature, flow-aligned chains tend to relax toward their isotropic state unless the flow stress is maintained, indicating that flow-induced orientational order alone is insufficient to promote rapid iPP nucleation in simulations. By inducing helical order in iPP chains via dihedral restraints, conformationally ordered chains quickly develop orientational order and crystallize in simulations at elevated temperatures. Extrapolation of the melting temperatures to zero restraint recovers experimental crystal melting behavior of iPP oligomers. Using classical nucleation theory, we show that conformational ordering can drastically reduce the nucleation barrier, whereas orientational alignment alone lowers it modestly, preventing nucleation on accessible MD timescales.

## Graphical abstract

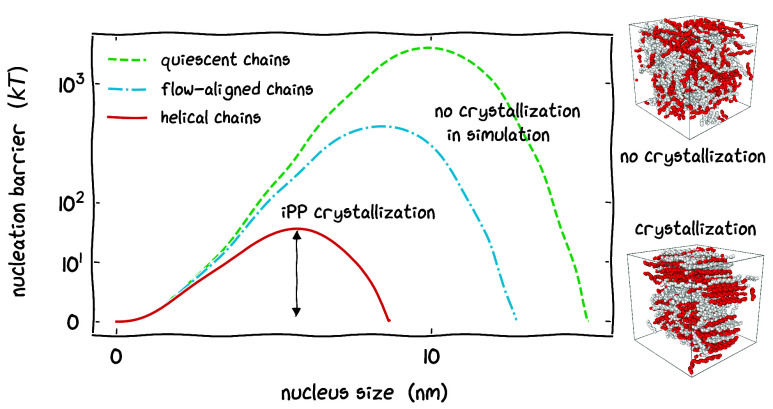

## I Introduction

Isotactic polypropylene (iPP) is a semicrystalline polymer^1,2^ well-known for crystallizing into a characteristic 3/1 helical structure, in which three monomer units complete one helical turn.^1,3,4^ The Rotational Isomeric State (RIS) method^1,5^ predicts that the entropy change during crystallization is primarily governed by the loss of conformational degrees of freedom associated with the coil-to-helix transition, while the enthalpy change arises largely from orientation-dependent interchain interactions.^1,6,7^ Thus, both conformational and orientational ordering are expected to play critical roles in iPP crystallization.^8–11^

By stretching and aligning polymer chains, orientational flows, such as shear^12–21^ and extensional flows,^9,10,19,22–26^ are known to enhance the crystallization kinetics of iPP. Despite extensive experimental evidence, the molecular pathway connecting flow-induced orientational alignment to the formation of helical conformations remains unresolved. In particular, it is unclear whether orientational order alone can drive chains into the specific conformational states required for nucleation, or whether intrinsic molecular factors—such as torsional barriers, conformational preferences, or steric constraints—play a dominant role.^9,10,27^ Heterogeneous nucleation has also been reported in the flow-induced crystallization (FIC) literature. While some studies found little influence from additives or nucleating agents,^12^ others observed strong enhancements in crystallization when such species were present, with the effect depending sensitively on their chemistry and concentration.^2,27,28^ More recently, Jacob et al.^29^ showed that high concentrations of foreign particles originating from catalyst residues can substantially accelerate nucleation. For broader historical context and comprehensive discussions of FIC, we refer readers to the reviews by Janeschitz-Kriegl^27^ and Nie et al.^30^

Molecular simulations can provide microscopic insights into the crystallization mechanisms of semicrystalline polymers.^31–33^ For simple nonhelical polymers such as polyethylene (PE), crystallization occurs rapidly enough to be captured within the timescales of current molecular dynamics (MD) simulations.^31,32^ Numerous studies have investigated the nucleation and crystal growth of PE oligomers.^32–40^ In contrast, crystallization of helical polymers like iPP remains challenging to capture in MD simulations due to their much slower kinetics.^11,31,33,41^ As a result, most research on iPP crystallization to date relies on experimental techniques.^2,42^ Previous computational studies have focused on predicting melting points of different iPP molar masses and exploring alternative methods to infer crystallization behavior,^11,41,43^ but a direct, molecular-level understanding of how iPP transforms from a melt state into its helical crystalline form is still lacking.^11,31,33^

In this work, we employ atomistic nonequilibrium molecular dynamics (atomistic NEMD) simulations to investigate the crystallization of iPP oligomers under two types of orientational fields: shear and uniaxial extensional flows. These flows are homogeneous and designed to mimic experimental setups, allowing us to probe their effects on helical formation and iPP crystallization. Our steady-state flow conditions differ from those used in previous nonequilibrium MD studies, which applied fast tensile deformation of the simulation box to emulate extensional flow.^44–46^ We show that while orientational flows enhance intermolecular alignment, they do not induce helical chain formation or crystallization. Upon quenching to room temperature, the flow-aligned chains—regardless of alignment degree—do not crystallize within accessible simulation times.

We demonstrate that conformational order is essential for iPP crystallization by inducing helical conformations in atomistic simulations. Using dihedral restraints, helically ordered chains tend to separate from coiled counterparts and crystallize. The critical temperature for iPP crystallization increases with the strength of the biasing potential. Extrapolating the phase transition temperatures to the limit of zero dihedral restraint recovers the melting temperatures of iPP oligomers with different molecular weights.

Using classical nucleation theory, we estimate the effects of intermolecular alignment and helical conformation on the nucleation barrier. Conformational ordering is far more effective at lowering the barrier and triggering nucleation in simulations. Overall, our findings highlight the critical role of conformational order in the nucleation and growth of iPP crystals. We expect that processing flows alone may be insufficient to induce the coil–helix transition in molten iPP, with the development of conformational order constituting the rate-limiting step in iPP crystallization.

## II Methods

### II.1 Preparing Molten iPP Oligomers

We prepare samples of molten iPP oligomers, each consisting of 400 chains, using GROMACS 2023.3 ^47^ at three different temperatures 400, 450, and 500 K. These systems represent iPP chains with 18, 30, and 60 monomers, which we denote as iPP18, iPP30, and iPP60, respectively. Initial configurations are generated using the *Polymer Builder* module in CHARMM-GUI.^48,49^ We employ the OPLS-AA force field^50^ to model iPP, as it accurately reproduces the melting temperatures of iPP with various molecular weights, as well as thermodynamic properties and crystal lattice constants.^43^ Each iPP system is equilibrated under NPT conditions using Berendsen barostat^51^ at 1 bar with a time coupling constant of 2 ps, and the stochastic velocity rescaling thermostat^52^ with a time constant of 0.1 ps at each temperature. The equilibration times exceed the Rouse relaxation times (see Table 1). Unless otherwise specified, we conduct postequilibrium simulations using Parrinello–Rahman barostat^53^ at the same pressure but with a coupling time of 20 ps, while maintaining the same thermostat setup. We use a 2 fs integration time and apply periodic boundary conditions in all directions.

**Table 1:** Rouse Relaxation Times, *τ*_R_, in Nanoseconds for the iPP Systems at Different Temperatures in Kelvin

relaxation time, τ_R_ (ns)				
system/temperature (K)	300	400	450	500
iPP18	360.1 ± 1.7	2.1 ± 0.1	0.8 ± 0.02	0.4 ± 0.01
iPP30	1000.3 ± 4.7^a^	10.7 ± 0.1	3.3 ± 0.03	1.4 ± 0.01
iPP60	4001.1 ± 18.9^a^	58.7 ± 0.7	18.7 ± 0.2	7.7 ± 0.1

a Indicates an extrapolation via the Rouse model scaling law (eq 2) with a first-order approximation of the error.

For iPP oligomers at different temperatures, we compute the longest Rouse relaxation time, τ_R_, from simulations of equilibrated melts as

1 $$\left\langle R\left(t\right)\cdot R\left(0\right)\right\rangle =\frac{{\left\langle R_{i}\left(t+\tau \right)\cdot R_{i}\left(\tau \right)\right\rangle }_{i,\tau }-{\left\langle R_{i}\right\rangle }_{i,\tau }^{2}}{{\left\langle R_{i}\left(0\right)\cdot R_{i}\left(0\right)\right\rangle }_{i,\tau }-{\left\langle R_{i}\right\rangle }_{i,\tau }^{2}}\approx \exp\left(-\frac{t}{\tau_{R}}\right)$$

where **R**_*i*_ is the end-to-end vector of polymer chain *i*, and ⟨ ⟩_*i*,τ_ denotes averaging the correlation over all chains and initial times τ. The corresponding values of τ_R_, which characterizes the autocorrelation time of the polymer end-to-end vectors,^1,54,55^ are listed in Table 1.

Because the equilibrated molten chains are later quenched to 300 K to study iPP crystallization, we also compute the characteristic relaxation time of the oligomers at this temperature. Since eq 1 did not converge for iPP30 and iPP60 after 3 μs at 300 K, we estimate their relaxation times using the Rouse scaling law,^1,55^

2 $$\tau_{R_{i}}=\tau_{R_{j}}\;{\left(\frac{N_{i}}{N_{j}}\right)}^{2}$$

where $\tau_{R_{j}}$ is the known relaxation time for chains of length *N*_*j*_ (iPP18 in our case), and *N*_*i*_ is the degree of polymerization for the longer chain whose relaxation time $\tau_{R_{i}}$ is being estimated.

### II.2 iPP under Shear and Uniaxial Extensional Flows

We apply simple shear and uniaxial extensional flows to induce alignment in molten iPP oligomers. These deformations are conducted at various strain rates ε̇ :

3 $$\begin{array}{ll} {\mathrm{\varepsilon ̇}}_{\mathrm{shear}} & =\left|\frac{\partial v_{x}}{\partial z}\right|,\\ {\mathrm{\varepsilon ̇}}_{\mathrm{uniaxial}} & =-\left|\frac{\partial v_{x}}{\partial x}\right|=-\left|\frac{\partial v_{y}}{\partial y}\right|=\frac{1}{2}\left|\frac{\partial v_{z}}{\partial z}\right| \end{array}$$

where v_*j*_ denotes the streaming velocity along the *j*-direction, and the strain rates above define the nonzero components of the velocity gradient tensor, ${\left(\nabla v\right)}_{ij}=\partial_{i}v_{j}$ .^56,57^ In shear flow, we impose a gradient of the velocity *x*-component, v_*x*_, along the *z*-direction (Figure 1(a)). In uniaxial extensional flow, we compress the system along the *x*- and *y*-directions while extending it along the *z*-direction (Figure 1(b)). The shear flow induces a local rotation of the polymer melt in the *xz*-plane, characterized by the vorticity $\omega =\nabla \times v=\left(0,\partial v_{x}/\partial z,0\right)$ ,^56,58,59^ whereas the uniaxial extensional flow has zero vorticity, ω = **0**.

**Figure 1:**
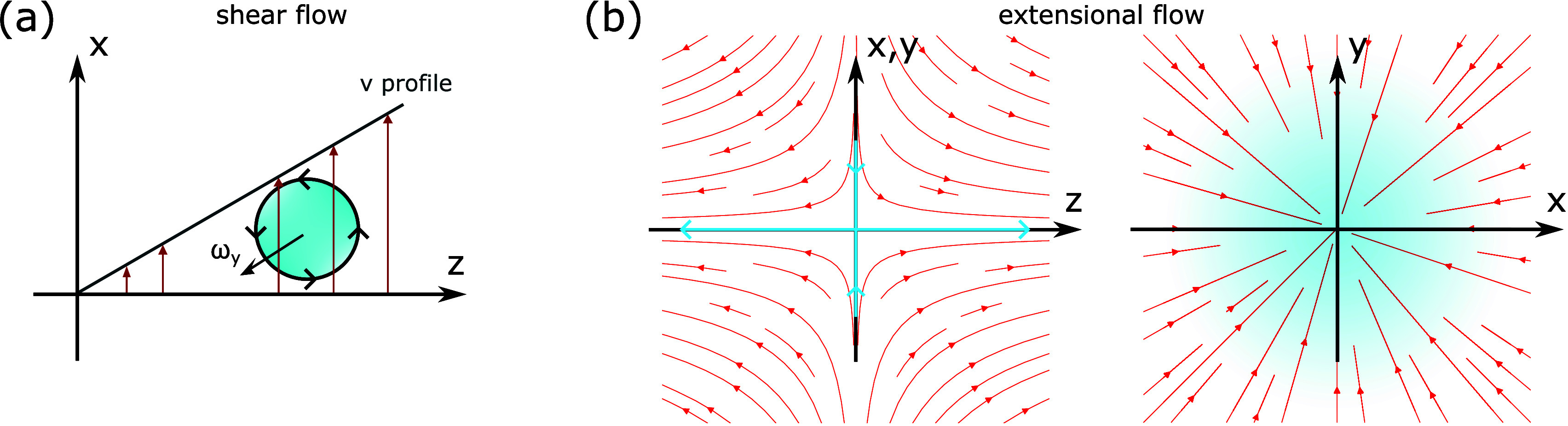
Schematic representations of (a) shear flow and (b) uniaxial extensional flow. In (a), velocity vectors vary linearly along the *z*-axis (dark red arrows), generating local rotational motion about the *y*-axis, quantified by the vorticity component ω_*y*_. In (b), streamlines (red arrows) show stretching along the *z*-axis and compression along the *x*- and *y*-axes (blue arrows). Strain rates are defined in eq 3.

The effective strength of the flow fields acting on iPP oligomers of different molecular weights is characterized by the dimensionless Weissenberg number *W*_*i*_:

4 $$W_{i}=\left|\mathrm{\varepsilon ̇}\right|\;\tau_{R}$$

where τ_R_ is the Rouse time (see Table 1). When *W*_*i*_ > 1, the flow fields outpace the conformational relaxation of the polymer chains, resulting in backbone stretching.^60–62^ Table S1 in the Supporting Information (SI) summarizes the strain rates and the corresponding *W*_*i*_ values for the iPP systems at different temperatures.

In our flow simulations, the equations of motion follow the SLLOD formalism^63–66^ and are carried out in LAMMPS (version 17 Apr 2024).^67^ We use the UEF package, developed by Nicholson and Rutledge,^68^ to perform simulations of iPP under uniaxial extensional flows, which—by construction—can be sustained indefinitely. Shear flows are coupled to a Nosé–Hoover barostat^69,70^ at 1 bar (≈0.987⁡atm) with a damping time of 2 ps along the vorticity direction (*y*-axis), which represents the free surface in a shear experiment. In uniaxial extensional flows, the same barostat is applied isotropically along the compressed coordinates (*x*- and *y*-axis), corresponding to the free surfaces in a typical drawing process along the *z*-axis. A Nosé–Hoover thermostat with a damping parameter of 0.1 ps is used to maintain the target temperature under both flow conditions. We run the simulations for 25 ns (iPP18 and iPP30) and 50 ns (iPP60), which is sufficient to reach steady states. Only for the iPP60 oligomers at 400 K and *W*_*i*_ = 1, we use the steady-state configuration at 500 K (with the same *W*_*i*_) as the initial configuration to accelerate reaching a steady state.

### II.3 Orientational Order Parameter for iPP

To measure the uniaxial alignment of the iPP chains, we compute the largest eigenvalue *q* of the orientational order tensor **Q**, defined as^39,66^

5 $$\left\langle Q\right\rangle =\frac{3}{2}{\left\langle t_{i}⊗t_{i}\right\rangle }_{i}-\frac{1}{2}I$$

where **t**_*i*_ is the unit vector across 3 successive iPP monomers, ⟨ ⟩_*i*_ denotes an ensemble average over all such vectors, ⊗ represents the outer product, and **I** is the 3 × 3 identity matrix.

The order parameter *q* ranges from 0 (isotropic) to 1 (perfect uniaxial order) and is equivalent to the second-order Legendre polynomial ${\left\langle P_{2}\left(t_{i}\cdot n\right)\right\rangle }_{i}$ used by other authors,^11,41,44,46^ except that it does not require specifying a reference director **n**. Unless stated otherwise, we compute *q* using all polymer chains.

### II.4 Inducing Conformational Order in Molten iPP

As we demonstrate later, fast flows can induce orientational order in polymer segments but do not promote the formation of conformational order, which is essential for iPP crystallization. To probe the role of conformational ordering, we restrain the backbone dihedral angles ϕ of half of the iPP chains (200 in total) in the melt using GROMACS. Specifically, we convert 100 chains into right-handed (RH) helices and 100 into left-handed (LH) helices by applying a restraining potential:

6 $$V_{dihr}=\frac{1}{2}\kappa_{dihr}\underset{l}{\sum }{\left(\phi_{l}-\phi_{l,0}\right)}^{2}$$

where κ_dihr_ is a positive dihedral force constant, ϕ_*l*_ is the dihedral angle *l*, ϕ_*l*,0_ is its reference value, and the sum runs over all dihedrals in both right-handed (RH) and left-handed (LH) chains. Adjusting κ_dihr_ tunes the degree of conformational ordering in the biased chains. For the RH chains, ϕ_*l*,0_ alternates between π (*trans* or *T*) and −π/3 (*gauche* ^–^ or *g*), while for LH chains, it alternates between π and π/3 (*gauche* ^+^ or *G*).^3,71^ We note that this definition is recommended by IUPAC^71^ and is opposite to the one adopted by Yamamoto^44^ and Sigalas et al.^46^ Even with a sufficiently strong restraining potential, the dihedral angles of helical chains still fluctuate around the ideal values ϕ_*l*,0_. Therefore, we assign a backbone dihedral within $\left(\phi_{l,0}\pm \pi /6\right)$ to a rotational isomeric state (RIS; see Table 2), according to IUPAC.^71^

**Table 2:** Ranges of Dihedral Angles for Each Rotational Isomeric State (RIS) of iPP

state	ideal angle ϕ_*l*,0_	dihedral range
*trans* (*T*)	π	(−π,−5π/6]⁡∪⁡[5π/6,π] ^a^
*gauche* ^–^ (*g*)	–π/3	[−π/2,−π/6] ^b^
*gauche* ^+^ (*G*)	π/3	[π/6,π/2] ^b^

a Antiperiplanar range.^44,46,72^ The symbol ∪ denotes the union of two sets.

b (±) Synclinal range.^44,46,72^

We quantify the number of helical turns based on the RIS states of successive backbone dihedrals: a *Tg* pair corresponds to a RH helix, while a *TG* pair indicates a LH helix. The relationship between the number of dihedrals and the number of helical turns is detailed in Sec. II of the SI, where we show that each *Tg* or *TG* pair tends toward 1/3 of a helical turn in a long polymer chain. In iPP, a complete helical turn consists of 3 consecutive helical monomers with the same handedness.

By counting the successive *Tg* and *TG* pairs, we compute both the number of dihedral pair groups and their sizes. As an example, consider the iPP18 chain shown below:
7

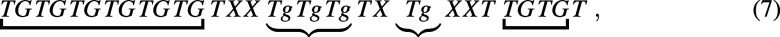

where *X* represents dihedrals that are not in the *T*, *G* or *g* states listed in Table 2. In this chain, there are two groups of dihedral pairs corresponding to each type of helix (*Tg* for RH and *TG* for LH). For iPP18, the number of helical turns per dihedral pair is 0.375 (see Sec. II of the Supporting Information), giving mean lengths of 0.75 and 1.5 turns for RH and LH helices, respectively. To quantify the conformational order in iPP samples, we compute the number-averaged mean length of helical turns per chain ⟨*N*_turns_⟩, hereafter referred to as the number-averaged helical turns.

## III Results and Discussion

### III.1 iPP in Extensional and Shear Flows

Fast extensional flows $\left(W_{i}>1\right)$ can effectively align polymer segments. To quantify the uniaxial alignment, we compute the steady-state nematic order parameter *q* as defined in Sec. II.3. The steady-state alignment increases with increasing *W*_*i*_ and eventually plateaus (Figure 2(a) and (b)). As the molar mass of iPP increases, the polymer chains exhibit a higher degree of uniaxial ordering, reflected in larger *q* values. There is no significant difference in alignment between 400 and 500 K when analyzed as a function of the dimensionless Weissenberg number *W*_*i*_. This is expected, as *W*_*i*_ maintains equivalent ratios of strain rate to relaxation time, leading to comparable deformation-relaxation dynamics across temperatures. However, because the relaxation time decreases with increasing temperature (see Table 1), achieving the same alignment at higher temperatures requires higher strain rates—and thus more energy input—even though the system reaches steady state more quickly (Sec. I of the SI).

**Figure 2:**
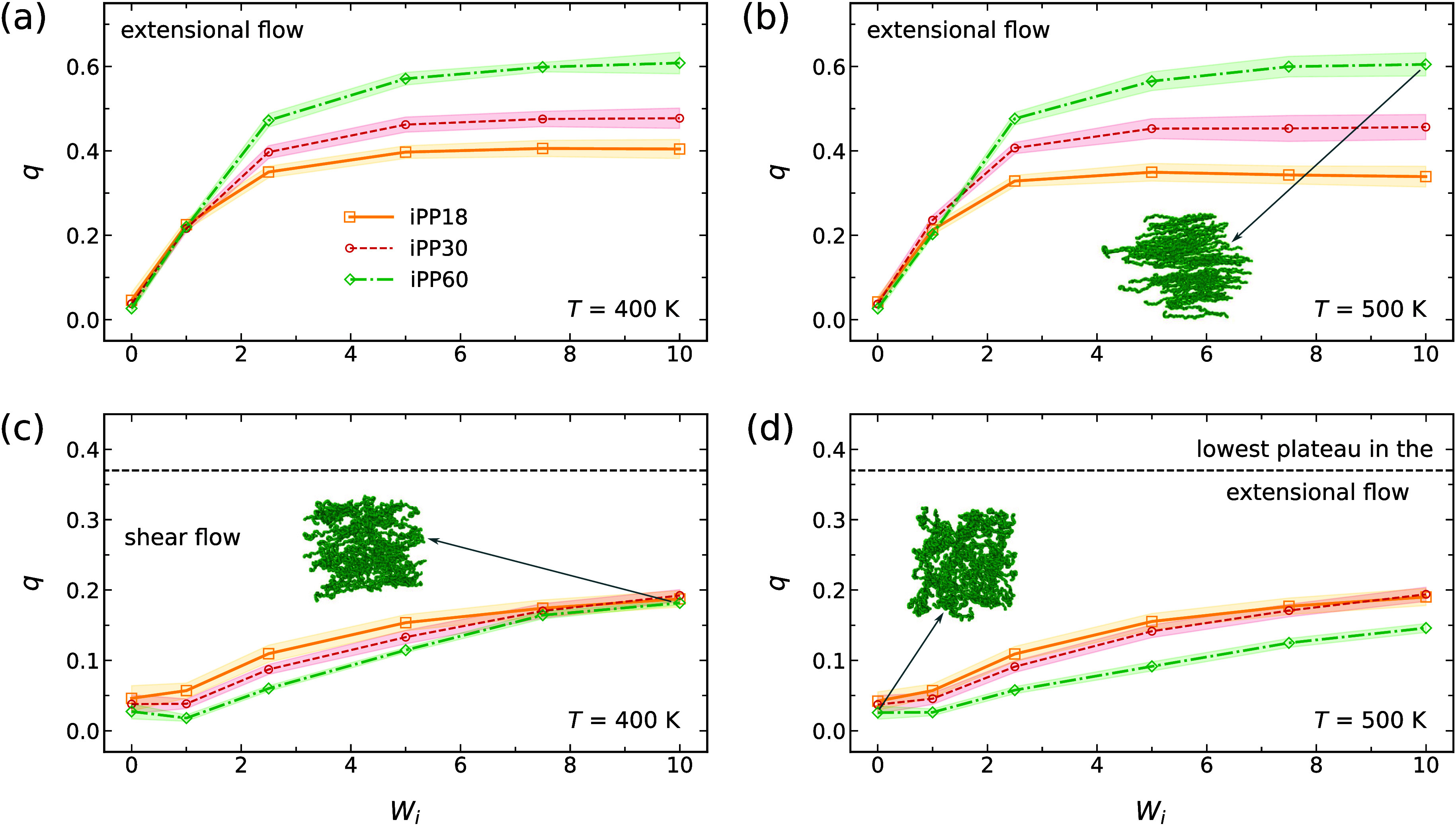
Steady-state nematic order parameter *q* vs *W*_*i*_ for iPP in extensional flows at (a) 400 K and (b) 500 K, and in shear flows at (c) 400 K and (d) 500 K. Insets: snapshots of some iPP60 backbone chains. Shaded regions indicate standard deviations.

Shear flows induce weaker orientational alignment compared to extensional flows. Owing to the presence of vorticity, the degree of uniaxial ordering *q* in shear flows (Figure 2(c) and (d)) is lower than in extensional flows (Figure 2(a) and (b)). This weaker alignment has also been observed experimentally. Stadlbauer et al.,^23^ Hadinata et al.,^73^ and Smith et al.^74^ reported that extensional flows align polymer chains more effectively than shear flow. As with extensional flows, there is no significant difference in alignment between 400 and 500 K since *W*_*i*_ values ensure comparable deformation-relaxation dynamics across temperatures. However, unlike extensional flows, shear flows show no clear dependence on molar mass.

The flow-induced order can be modeled by introducing an Onsager-style trial orientational distribution function of polymer segments:^75^

8 $$f\left(\theta \right)=\frac{A}{4\pi }\frac{\exp\left(A\cos\theta \right)}{\sinh A}$$

where *A* is a variational parameter used to minimize the orientational free energy. Subbotin and Semenov^76^ applied this trial function to construct a variational mean-field free energy, showing that the orientational order of polymer segments under extensional flow in dilute solution depends on both the strain rate and the number of monomers *N*.

Instead of executing a similar variational approach, we determine *A* as a function of *W*_*i*_ and chain length directly from our simulations. To do so, we first express the average orientational order parameter as

9 $$\begin{array}{ll} q & =\int_{0}^{2\pi }d\phi \int_{0}^{\pi }P_{2}\left(\cos\theta \right)\;f\left(\theta \right)\sin\theta d\theta \\ & =1-\frac{3}{A}\coth A+\frac{3}{A^{2}} \end{array}$$

For *A* → 0, q(A)=0 , corresponding to an isotropic distribution, while for *A* → *∞*, q(A)=1 , indicating perfect uniaxial alignment.

From the orientational order parameter *q* as a function of *W*_*i*_, obtained from simulations at 400, 450, and 500 K, the corresponding *A* values are determined using eq 9 for both extensional and shear flows under steady-state conditions. Similar to dilute polymer solutions,^76^ the extracted *A* values for molten iPP under extensional flow depend on the number of monomers *N*. In contrast, no clear *N*-dependence is observed for molten iPP under shear flow. By fitting these *A* values, we show

10 $$\begin{array}{ll} A & =\frac{b_{1}W_{i}\;N^{1/2}}{b_{2}W_{i}+N^{1/2}},\left(\mathrm{extensional}\mathrm{flows}\right),\\ A & =\frac{c_{1}W_{i}}{c_{2}+W_{i}},\left(\mathrm{shear}\mathrm{flows}\right) \end{array}$$

where *b*_1_ = 3.83 ± 0.03 and *b*_2_ = 3.97 ± 0.04, and *c*_1_ = 2.29 ± 0.01 and *c*_2_ = 2.21 ± 0.05 are the fitting parameters for the iPP systems, reported with their standard errors. When substituted into eq 9, these expressions closely reproduce the simulation data, as displayed in Figure 3. Sections III and IV of the SI provide flow-induced order *q* at 450 K for different iPP oligomers, showing trends consistent with other temperatures when plotted as a function of *W*_*i*_. Our fitting results may help estimate the segmental alignment *q* for iPP of different lengths stretched by fast orientational flow with *W*_*i*_ > 1.^60–62^

**Figure 3:**
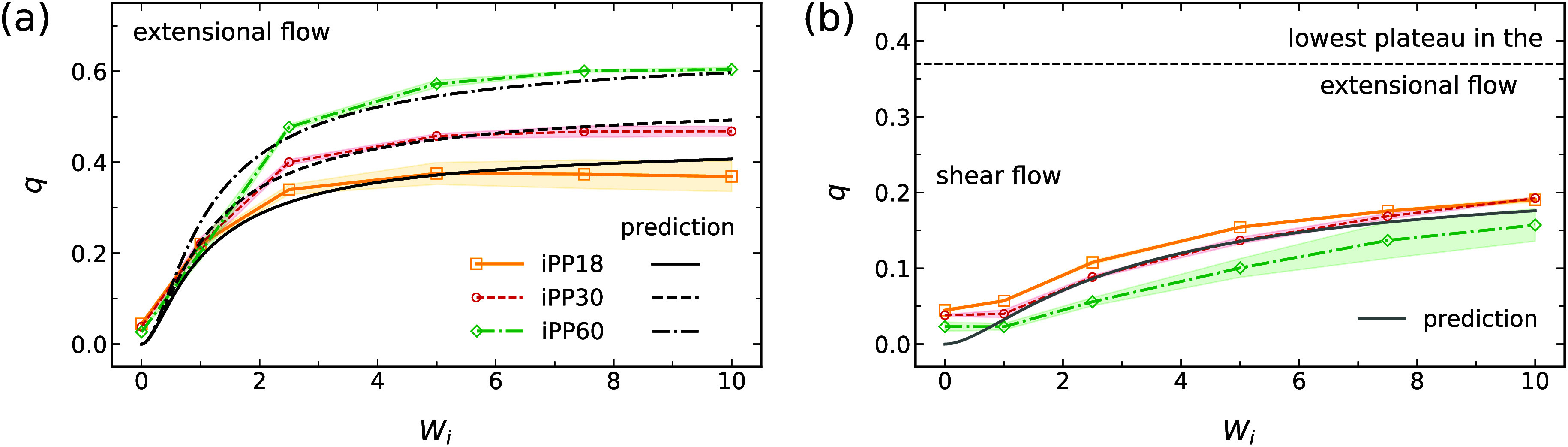
Predicted nematic order *q* vs *W*_*i*_ for (a) extensional and (b) shear flows using eqs 9 and 10 (black and gray curves). Simulation data averaged over different temperatures (colored symbols and curves). Shaded regions indicate standard deviations.

Both extensional and shear flows, however, do not induce significant conformational order in iPP. In extensional and shear flows, the number-averaged helical length remains below one full turn, with negligible variation across different *W*_*i*_ values (Figure 4). This indicates that while uniaxial extensional and shear flows can promote uniaxial alignment of polymer chains, they do not induce intramolecular conformational changes. The slight differences in ⟨*N*_turns_⟩ between temperatures are attributed to thermal fluctuations, with larger fluctuations at 500 K resulting in fewer helical turns. In Sections III and IV of the Supporting Information (SI), we provide data at 450 K, including separately reported counts of right-handed (RH) and left-handed (LH) helices, which are statistically equivalent.

**Figure 4:**
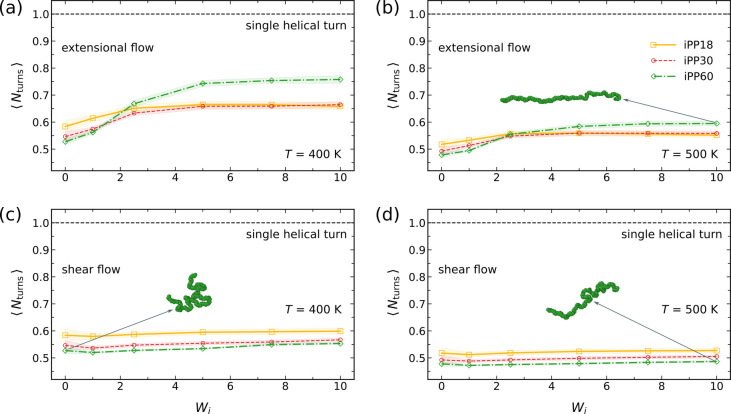
Number-averaged helical length ⟨*N*_turns_⟩ of iPP oligomers under extensional flows at (a) 400 K and (b) 500 K, and under shear flows at (c) 400 K and (d) 500 K. Insets: snapshots of single iPP60 backbone chains. Shaded regions indicate standard deviations.

Our results differ somewhat from earlier studies in which fast uniaxial tensile deformation was used to stretch iPP chains.^44–46^ These studies showed that fast tensile deformation can align polymer chains and slightly enhance helical order in iPP at large applied strains. For example, in a united-atom iPP model consisting of 2000 monomers, the weight-averaged helical segment length was reported to reach about 8 monomers ( ≈2.67 helical turns) before cavities began to form under large tensile stress.^46^ We attribute the discrepancy to differences in the flow conditions and in the definition of the conformational states. By simulating iPP18 under extensional flow using an alternative all-atom force field (COMPASS^77^), as employed by Xie et al.,^45^ we find that the choice of force field (OPLS-AA or COMPASS) does not affect our results (see Sec. V of the SI for details). Isotactic polypropylene under steady-state extensional flow may behave differently from chains subjected to transient and fast tensile deformation. Additionally, we used narrower dihedral angle intervals to define *T*, *g* and *G* conformations (see Table 2). Relaxing these RIS criteria can lead to higher helical order in molten iPP. For example, Xie et al.^45^ employed broader dihedral ranges to define helical turns and consequently reported more helices than Yamamoto^44^ and Sigalas et al.^46^ In Sec. VI of the SI, we compare our IUPAC-based dihedral-angle intervals with the broader definition from ref 45. The broader definition of helical conformation only results in a slight increase in helical segments. Overall, iPP oligomers do not develop conformational order in simulations under fast shear and extensional flows.

### III.2 Quenching Flow-Aligned iPP Samples

We quench samples with the highest flow-induced alignment—prepared in extensional flows at *W*_*i*_ = 10 and 500 K—to 300 K, which is below the experimental melting point of all the iPP oligomers studied (see Table 3). Two scenarios are considered: (1) continuing the uniaxial extensional flow by maintaining the same *W*_*i*_ at 300 K, and (2) turning off the flow to investigate the relaxation behavior of the polymer segments.

**Table 3:** Comparison of Melting Temperature $T_{m}^{0}$ in Kelvin (K) for Each iPP System

$T_{m}^{0}$ (K)	iPP18	iPP30	iPP60	iPP∞^e^
simulation (this work^a^)	330 ± 10	370 ± 5	398 ± 3	428 ± 2
simulation^b^ (ref 43)	343 ± 6	391 ± 6	428 ± 5	464 ± 12
simulation^c^ (refs 11 and 41 )	376	389		
experiment (ref 43)	323	379	422	462 ± 18
experiment^d^ (ref 79)	341	381	412	426 ± 8

a Using eq 12 and data in Figure 7(b).

b Average temperature for melting iPP crystals from vacuum-crystal interfaces along the (010) and (100) directions.

c The data in ref 41 for iPP17 complement those in ref 11. Both studies employ the same method to estimate $T_{m}^{0}$ . We report a linear interpolation estimate.

d Linear interpolation of the purified and fractionated solid isotactic polypropylene (iPP) data, which corresponds to fractions F–J in ref 79.

e The bulk melting temperature obtained by linearly extrapolating the $T_{m}^{0}$ versus 1/*MM* plot to infinite molar mass *MM* → ∞.^43^

In extensional flows at the same *W*_*i*_, the nematic order parameter *q* of iPP at 300 K remains comparable to that resulting from the prior flow history at 500 K, as shown in Figure 5(a). Because the relaxation time increases as temperature decreases (see Table 1), the strain rate at 300 K is lower than at 500 K (Sec. I of the SI). A slight transient relaxation of the prealigned chains is initially observed in Figure 5(a), as the system adjusts to the new flow conditions before reaching a new steady state.

**Figure 5:**
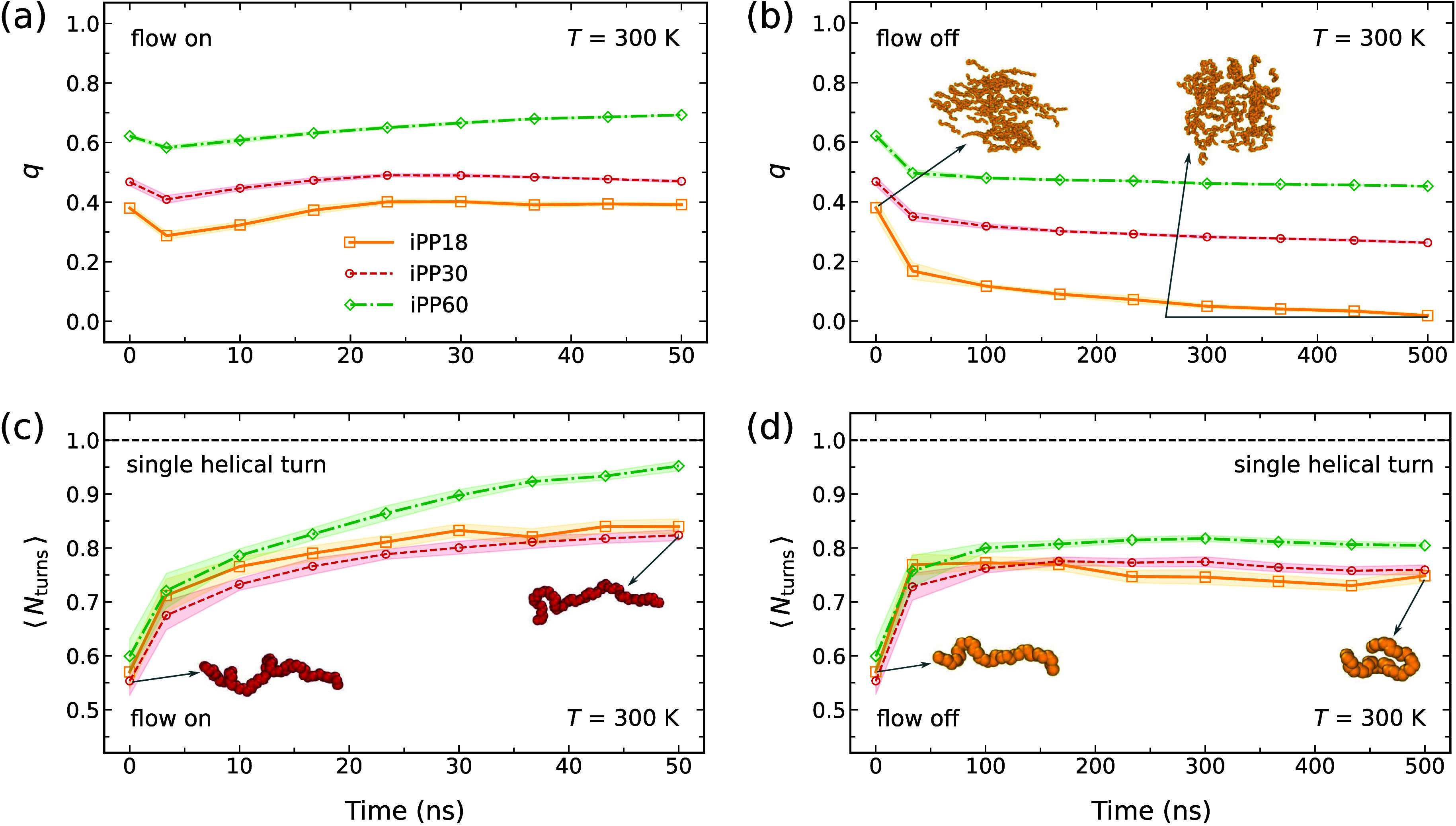
Nematic order parameter *q* vs time at 300 K for samples in (a) extensional flow and (b) without applied flow. Average number of helical turns ⟨*N*_turns_⟩ for samples in (c) extensional flow and (d) without flow. Insets show snapshots of iPP backbone oligomers: iPP18 (yellow) and iPP30 (red). Shaded regions indicate standard deviations.

In contrast, when the strain rate is completely removed, the system is unable to maintain its orientational order, as shown in Figure 5(b). In the absence of flow (Figure 5(b)), the order parameter *q* exhibits an initial rapid decay followed by a slower relaxation as the systems return to the isotropic configuration. The relaxation depends on the iPP molar mass, with higher molar-mass samples retaining their flow history for longer periods.

Under both flowing and nonflowing conditions, the number of successive helical turns remains below one in our simulations. In extensional flows, the supercooled melts slowly develop conformational order over time (Figure 5(c)). Without external flows, iPP oligomers exhibit slight enhancements in helical segment length due to reduced thermal fluctuations at the quenched temperature (Figure 5(d)). The counts of RH and LH helical turns are provided in Sec. VII of the SI, along with a comparison between OPLS-AA and COMPASS for a highly aligned iPP18 sample after flow cessation and quenching to 300 K. No qualitative differences are observed between the two force fields. Overall, the uniaxially aligned iPP samples below their melting temperatures do not crystallize within any practical simulation time. Even in highly aligned samples, nucleating conformational order in simulations remains challenging.

### III.3 Conformational Ordering

We expect conformational ordering to be the rate-limiting step in iPP crystallization. To show this, we apply dihedral restraints, which promote helical ordering, to iPP chains under quiescent conditions (see Sec. II.4). By restraining half of the polymer chains (100 RH and 100 LH helices), the induced conformational order promotes fast iPP crystal nucleation from isotropic melts in simulations (Figure 6). The strength of the dihedral restraints is insufficient to produce all-helical rods. The semiflexible helical iPP can relax via translational and rotational diffusion in “theta solvents” that consist of unrestrained iPP chains. Romanos et al.^11,41^ similarly induced iPP crystallization by promoting helix formation through restraints that stretched every three monomers to the length of a full helical turn.

**Figure 6:**
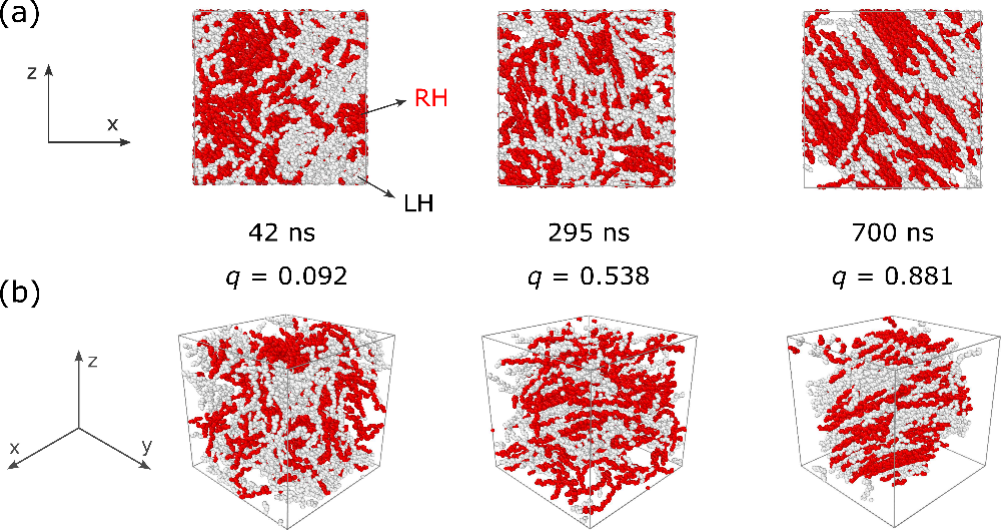
Snapshots of conformationally ordered iPP30 at 500 K: (a) *xz*-plane view and (b) perspective view of chain backbones. Averaged orientational order *q* of helical chains listed for each snapshot. Right-handed (RH) chains (red) and left-handed (LH) chains (white). Coiled chains are omitted for clarity. Dihedral force constant is κ_dihr_ = 15 kJ mol^–1^ rad^–2^ (eq 6), which yields ⟨*N*_turns_⟩ = 1.89 at 700 ns for the restrained chains.

The enhanced crystallization kinetics arise from a stronger thermodynamic driving force. Increasing the degree of conformational ordering of molten chains reduces the entropy of fusion, thus raising the melting temperature.^11,41,78^ To estimate the melting temperatures *T*_m_ of chains with preimposed helical order, we melt single crystals prepared with varying dihedral force constants κ_dihr_ (eq 6), using an annealing rate of 1 K/ns. This rate has previously been shown to yield melting temperatures and thermodynamic properties of iPP oligomers in good agreement with experimental results.^43^ During the temperature ramp, we monitor the orientational order *q* of the restrained chains (Figure 7(a)).

**Figure 7:**
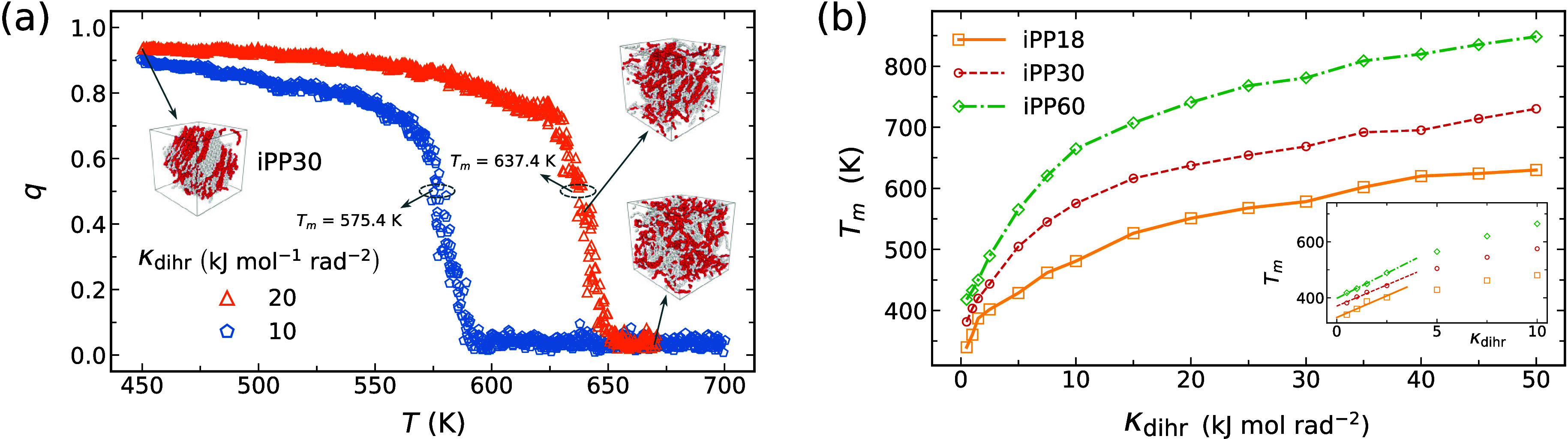
(a) Orientational order *q* vs temperature *T* during temperature ramping for iPP30 with two different dihedral restraints κ_dihr_. Insets: snapshots of restrained chains. (b) Melting temperature *T*_m_ vs κ_dihr_ for the iPP samples. Inset: linear extrapolations to κ_dihr_ = 0 for estimating the melting temperatures $T_{m}^{0}$ . Standard deviations smaller than the markers and omitted for clarity.

To better extract *T*_m_, we assume the order parameter *q* “equilibrates” at each temperature *T* and model its temperature dependence using a two-state model, consisting of a lower-energy crystalline state and a higher-energy melt state separated by a free energy difference Δ*G*. From this, the average orientational order of the restrained iPP is

11 $$q=\frac{1}{\exp\left(-\beta \Delta G\right)+1}$$

where $\beta ={\left(kT\right)}^{-1}$ , with *k* being the Boltzmann constant. During the heating scan (Figure 7(a)), we determine the melting temperature *T*_m_ (Figure 7(b)) as the point where Δ*G* = 0, corresponding to *q* = 0.5 and indicating equilibrium between the crystal and melt phases.

The enhanced *T*_m_ arises from the reduced conformational entropy in the melt state,^11,41,78^ which we expect to be linear in κ_dihr_ when the applied restraint is weak. Thus, the melting temperature of helical iPP oligomers can be expressed as

12 $$\begin{array}{ll} T_{m} & =\frac{\Delta H_{f}}{\Delta S_{f}-\left|\alpha \right|\kappa_{dihr}}=\frac{\Delta H_{f}}{\Delta S_{f}}\left(\frac{1}{1-\frac{\left|\alpha \right|\kappa_{dihr}}{\Delta S_{f}}}\right)\\ & \approx \frac{\Delta H_{f}}{\Delta S_{f}}+\left(\frac{\Delta H_{f}}{\Delta S_{f}}\frac{\left|\alpha \right|}{\Delta S_{f}}\right)\kappa_{dihr}\\ & =T_{m}^{0}+\gamma \kappa_{dihr} \end{array}$$

where Δ*H*_f_ and Δ*S*_f_ are the enthalpy and entropy of fusion, respectively; the unknown constant |α| is the first-order coefficient for expanding the entropic penalty imposed by the dihedral restraint, and $T_{m}^{0}$ denotes the melting temperature when κ_dihr_ = 0. The second line of eq 12 employs the geometric series truncated to first order.^57^ To extrapolate $T_{m}^{0}$ for free iPP oligomers (see Figure 7(b)), we take *T*_m_ values for dihedral-restrained iPP within the linear regime (κ_dihr_ ≤ 2.5 kJ mol^−1^ rad^−2^). This linear regime, identical across the studied iPP oligomers, is identified by iteratively fitting linear models to subsets of data beginning with the lowest κ_dihr_ values, using residual analysis to confirm linearity.

The extrapolated melting temperatures of iPP without dihedral restraints $T_{m}^{0}$ are in good agreement with experimental data. Table 3 summarizes the melting temperatures for our iPP systems, alongside simulation and experimental values from previous studies. The value of $T_{m}^{0}$ obtained from eq 12 and Figure 7 agrees with both experimental and computational results, indicating that conformational ordering—rather than orientational ordering alone—is the primary driving force for crystallization. Because the single crystals in our simulations are finite and less ideal than those constructed in refs 11, 41, and 43 , our estimated melting temperatures are expected to fall slightly below their reported values. Variations in experimental values likely arise from differences in crystallinity or structural defects.

### III.4 Orientational and Conformational Contributions to the iPP Nucleation Barrier

To better illustrate the roles of orientational and conformational ordering in iPP crystal nucleation, we apply classical nucleation theory^80,81^ to estimate how different types of precursor ordering affect the nucleation barrier of iPP18 oligomers. For a cylindrical crystal nucleus with radius *R* and length *L*, the free energy is given by^81^

13 $$F\left(R,L\right)=\left(2\pi R^{2}\right)\gamma_{\mathrm{end}}+\left(2\pi RL\right)\gamma_{\mathrm{side}}+\left(\pi R^{2}L\right)\Delta g_{\mathrm{crystal}}$$

where γ_end_ and γ_side_ are the interfacial free energies of the nucleus-end and side (lateral) surfaces, respectively, and Δ*g*_crystal_ is the bulk free energy density difference between crystalline and molten chains. At 300 K, we use Δ*g*_crystal_ = −2.2 *kT*/nm^3^ for iPP18, as reported in ref 7 (see Figure S11 in the SI).

To estimate the interfacial free energies between crystalline and molten chains (γ_end_ and γ_side_), we model the melt-crystal interface using the one-dimensional Cahn–Hilliard interfacial functional:^82–84^

14 $$\gamma \left[q\right]=kT\int dz\left[\frac{\zeta }{2}{\left(\frac{\partial q}{\partial z}\right)}^{2}+wq^{2}{\left(1-q\right)}^{2}\right]$$

where ζ and *w* are positive constants with dimensions of inverse length and inverse volume, respectively. The order parameter q=q(z) varies across the flat interface between crystalline and molten domains. The gradient term in eq 14 penalizes spatial variations of *q*, ensuring a smooth, finite-width transition between phases, while the quartic term in *q* represents a symmetric “double-well” potential that accounts for the thermodynamic penalty of mixing, i.e., occupying an intermediate state between the two phases.^84^ Details about the values of ζ and *w* are provided in Sec. IX.1 of the SI. Briefly, ζ is chosen based on the iPP crystal structure, while *w* is obtained by fitting the iPP18 free energy density *g* near the melting temperature, where the crystal and melt are equally stable.

Minimizing the interfacial functional in eq 14, δγ[q]/δq=0 , gives the equilibrium profile of the crystal-melt interface:^85^

15 $$q\left(z\right)=\frac{1}{2}\left[1+\tanh\left(\frac{z}{2b}\right)\right]$$

where the interfacial width is *b* = (ζ/2*w*)^1/2^ (see Sec. IX.1 of the SI for details). Using q(z) , the interfacial free energies are computed as

16 $$\gamma =kT\frac{wb}{3}$$

At 300 K, γ_end_ and γ_side_ for iPP18 are 21.0 and 32.4 mN/m, respectively. Our estimated side interfacial free energy qualitatively agrees with the value 45.4 mN/m reported for iPP30, obtained from atomistic free energy calculations at a temperature above the iPP melting point.^86^

Assuming the interfacial free energy is independent of temperature, we estimate the critical dimensions of an iPP18 cylindrical nucleus at 300 K. Minimizing eq 13 with respect to each independent variable gives the critical radius R* and length L* for primary nucleation,

17 $$\begin{array}{ll} R^{*} & =-\frac{2\gamma_{\mathrm{side}}}{\Delta g_{\mathrm{crystal}}}=7.1\mathrm{nm},\\ L^{*} & =-\frac{4\gamma_{\mathrm{end}}}{\Delta g_{\mathrm{crystal}}}=9.2\mathrm{nm} \end{array}$$

which, when substituted into eq 13, gives the critical free-energy (nucleation) barrier:

18 $$F^{*}=\frac{8\pi {\left(\gamma_{\mathrm{side}}\right)}^{2}\gamma_{\mathrm{end}}}{{\left(\Delta g_{\mathrm{crystal}}\right)}^{2}}=1607kT$$

The critical nucleus size for iPP18 (eq 17) qualitatively agrees with experiments, and its large nucleation barrier indicates slow crystallization. Using iPP melt confined in aluminum oxide nanopores, Duran et al.^87^ did not observe iPP nucleation in pores with diameters smaller than 20 nm, providing an experimental estimate of the critical nucleus size. This is consistent with our critical nucleus size of approximately 10 nm. Experimentally, iPP crystallization occurs on the timescale of minutes,^42^ which may result from the large nucleation barrier. This slow nucleation is not accessible in MD simulations.

In the following, we estimate the effects of orientational and conformational ordering on the nucleation barrier of iPP18 at 300 K using eq 18. We assume that only Δ*g*_crystal_ depends on the imposed orientational and conformational order, while the interfacial free energies remain unchanged (see eq S7 in the SI). We note that the interfacial free energies are expected to decrease as the preordered melt phase becomes more similar to the crystal, which, according to eq 18, implies that our estimated nucleation barriers represent upper bounds. Quantifying the effects of orientational and conformational ordering on melt-crystal interfacial free energies is beyond the scope of this paper.

For orientational ordering, Δ*g*_crystal_ is expressed as a function of the order parameter *q*:

19 $$\Delta g_{\mathrm{crystal}}\left(q\right)=g\left(1\right)-g\left(q\right)$$

where the data for the free energy density g(q) come from ref 7 and are available in Figure S11 of the SI. For the conformational ordering, Δ*g*_crystal_ is expressed as a function of the number of helical turns *N*_turns_:

20 $$\Delta g_{\mathrm{crystal}}^{N_{\mathrm{turns}}}=\Delta g_{\mathrm{crystal}}\left(0\right)+T\Delta s_{\mathrm{crystal}}^{h}\left\langle N_{\mathrm{turns}}\right\rangle$$

where $\Delta g_{\mathrm{crystal}}\left(0\right)$ is defined in eq 19, and $\Delta s_{\mathrm{crystal}}^{h}$ denotes the crystallization entropy density per helical turn, reported in eq S9 of the SI for conciseness. This entropy term arises from reducing the available RIS from three to one as polymer segments transition from coil to helical, amounting to −*kT* ln 3 per backbone dihedral angle.^7^

Conformational ordering is more effective than orientational ordering at reducing the nucleation barrier of iPP18 (Figure 8). Orientational ordering can lower the critical nucleation barrier by up to an order of magnitude, yet it remains high even for highly aligned chains (large *q* values). By contrast, preimposed conformational ordering can reduce the nucleation barrier to the order of 10 *kT* for iPP18 oligomers at 300 K.

**Figure 8:**
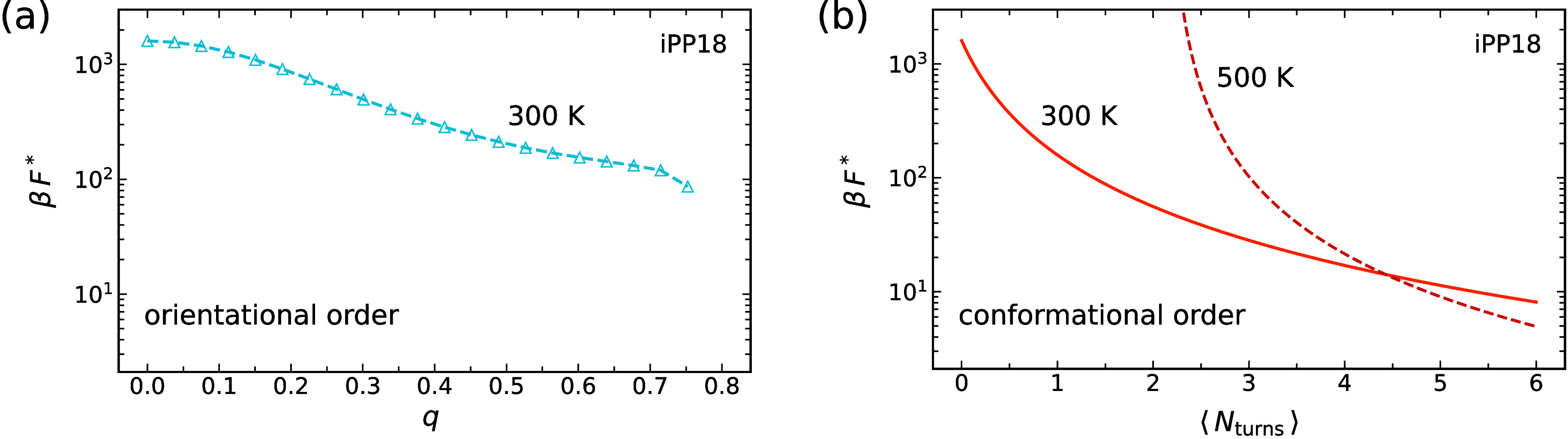
Nucleation barrier βF* for iPP18 vs (a) orientational order *q*, and (b) conformational order, quantified by the number of helical turns ⟨*N*_turns_⟩. Curves are shown for the indicated temperatures. For iPP18, maximum number ⟨*N*_turns_⟩ = 6.

We also estimate the nucleation barrier for iPP18 oligomers with preimposed helical order at 500 K, which is of order 10 *kT* (Figure 8(b)). For these chains, the free energy density difference between the crystal and melt Δ*g*_crystal_ is approximately 10.34 *kT*/nm^3^, assuming the equilibrium melting temperature of iPP18 oligomers is 320 K and the enthalpy of fusion is temperature independent (details are provided in Sec. IX.3 of the SI). The positive free energy density at this high temperature indicates that the crystallization of free iPP18 is not spontaneous. However, by reducing the conformational entropy of the molten chains (eq 20), the helically ordered chains can crystallize even at the relatively high temperature of 500 K. Using the γ_end_ and γ_side_ for free iPP18, we estimate that the nucleation barrier for the preordered helical chains is on the order of 10 *kT* at 500 K.

This difference between the nucleation-barrier reductions, induced by preimposed orientational and conformational order, explains why nucleation is not observed in our flow-aligned chains upon quenching (Sec. [Sec sec3.2]), whereas iPP nucleation and crystallization occur when helical ordering is induced in iPP oligomers (Sec. [Sec sec3.3]) even at higher temperatures. Our flow-aligned chains do not form helical segments, and while orientational alignment decreases the nucleation barrier, it remains too high for nucleation to occur on the accessible MD timescale.

## IV Conclusion

We use nonequilibrium atomistic MD simulations to investigate the roles of orientational and conformational ordering in the crystallization of iPP oligomers. Both extensional and simple shear flows promote uniaxial alignment, but neither induces helix formation. Extensional flow aligns chains more effectively, producing a flow-induced orientational order that depends on molecular weight and flow strength, characterized by the Weissenberg number *W*_*i*_. In contrast, shear flow produces weaker alignment with no dependence on chain length.

Upon quenching below the melting temperature and removing the flow stress, uniaxially ordered iPP chains gradually relax toward their isotropic state in simulations. This relaxation is slow and molar-mass dependent, with higher molar-mass samples taking longer. However, when the flow strength is maintained at the same *W*_*i*_, the chain orientation persists, reflecting the prior flow history. No iPP crystallization is observed in the flow-aligned samples.

Biasing the conformational order of iPP triggers helix formation and promotes crystallization in simulations. Extrapolating the melting temperatures to zero restraint is consistent with experimental data and prior simulation studies of free iPP oligomers, demonstrating that the development of conformational order is essential for iPP crystallization.

Using classical nucleation theory, we show that conformational ordering is more effective than orientational ordering in lowering the nucleation barrier of iPP18. Flow-induced orientational alignment can lower the nucleation barrier by an order of magnitude, but it remains too high for nucleation to occur within accessible MD timescales. On the other hand, imposing conformational order can drastically lower the barrier to around 10 *kT*, allowing iPP crystallization to be observed in simulations.

## Supplementary Material



## Notes

Additional details on the studied systems, including Weissenberg numbers, strain rate data, results for extensional and shear flows at 450 K, dihedral angle distributions, and estimates of the free energy barrier (PDF)
