# Supplementary material for: Effects of Orientational and Conformational Ordering on Isotactic Polypropylene Crystallization
Source: Macromolecules. 2026 Jan 14;59(2):962–73. doi: 10.1021/acs.macromol.5c03407 (PMC12854746; doi:10.1021/acs.macromol.5c03407)
Supplement: Supplementary file 1 [file ma5c03407_si_001.pdf]

Supplementary Information for "Effects of Orientational and Conformational Ordering on Isotactic Polypropylene Crystallization"

Anderson D. S. Duraes and Wenlin Zhang*

41 College Street, Department of Chemistry,

Dartmouth College, Hanover, New Hampshire 03755, United States

E-mail: wenlin.zhang@dartmouth.edu

Contents

I Weissenberg Number and Strain Rate **S3**

II Relationship between Dihedral Angles and Helical Turns **S4**

III Extensional Flows **S5**

IV Shear Flows **S7**

V Comparison Between Force Fields **S9**

VI Conformational Definitions: Broader Dihedral-Angle Ranges **S11**

VII Quenching iPP Samples **S12**

VIII Dihedral Angle Distribution Analysis **S14**

IX Orientational and Conformational Contributions to the iPP18 Nucleation Barrier **S15**

IX.1 Interfacial Free Energies S15

IX.2 Crystallization Entropy Density S17

IX.3 Estimation of Free Energy Density at 500 K S17

References **S19**

I Weissenberg Number and Strain Rate

Table S1 lists the strain rates ($\dot{\varepsilon}$) used in the non-equilibrium molecular dynamics (NEMD) simulations for the corresponding Weissenberg numbers W_i. These strain rates are calculated using Eq. (4) and the Rouse relaxation times from Table 1, both provided in the main text. As the number of monomers increases, the strain rates approach practical experimental values. [1]

Table S1: Strain Rates $(\dot{\varepsilon})$ in nanoseconds^{-1} $\left(\text{ns}^{-1}\right)$ Corresponding to the Considered Weissenberg Numbers (W_i) in the NEMD Simulations

$\boldsymbol{T = 300}$ K					
iPP / W_i	1	2.5	5	7.5	10
18	0.00278	0.00694	0.0139	0.0208	0.0278
30	0.001	0.0025	0.005	0.0075	0.01
60	0.000250	0.000625	0.00125	0.00187	0.00250
$\boldsymbol{T = 400}$ K					
iPP / W_i	1	2.5	5	7.5	10
18	0.476	1.19	2.38	3.57	4.76
30	0.0935	0.234	0.467	0.701	0.935
60	0.0170	0.0426	0.0852	0.128	0.170
$\boldsymbol{T = 450}$ K					
iPP / W_i	1	2.5	5	7.5	10
18	1.25	3.13	6.25	9.38	12.5
30	0.303	0.758	1.52	2.27	3.03
60	0.0535	0.134	0.267	0.401	0.535
$\boldsymbol{T = 500}$ K					
iPP / W_i	1	2.5	5	7.5	10
18	2.50	6.25	12.5	18.8	25.0
30	0.714	1.79	3.57	5.36	7.14
60	0.130	0.325	0.649	0.974	1.30

II Relationship between Dihedral Angles and Helical Turns

Isotactic polypropylene (iPP) has three rotational isomeric states along its backbone:[2, 3] *trans* (T), *gauche*$^{-}$ (g) and *gauche*$^{+}$ (G), whose ideal dihedral angles correspond to π, $-\pi/3$ and $\pi/3$, respectively. In this work, dihedral angles range from $-\pi < \phi \leq \pi$, and the states are assigned according to Table 2 in the main paper.

We quantify the number of helical turns based on adjacent dihedral pair states: Tg for right-handed (RH) helices and TG for left-handed (LH) helices. For an iPP backbone with N monomers, there are $2N$ carbon atoms and, consequently, $(2N-3)$ dihedral angles, since each dihedral corresponds to a unique sequence of four consecutive carbon atoms. This results in $(2N-4)$ adjacent dihedral pairs, where each pair spans two successive dihedrals and can be indexed by the super-diagonal of a matrix whose diagonal indices represent individual dihedrals. Among the $(2N-4)$ adjacent pairs, only $(2N-4)/2$ are non-overlapping and thus contribute independently to helical turns. Because each helical turn in iPP consists of 3 consecutive helical monomers, the maximum number of turns is $N/3$. Thus, the number of helical turns per non-overlapping dihedral pairs is

$$\frac{\text{helical turns}}{\text{dihedral pairs}} = \frac{N}{3} \cdot \frac{2}{2N-4}\,, \tag{S1}$$

which tends to $1/3$ in the limit of long iPP chains ($N \to \infty$).

For iPP18, the number of helical turns per non-overlapping dihedral pair is 0.375 (Eq. (S1)). In the iPP18 chain shown in Eq. (7) of the main paper, there are 2 Tg groups: one consisting of 3 consecutive pairs and another consisting of a single pair. This gives an average of 2 consecutive Tg pairs, resulting in $2 \times 0.375 = 0.75$ RH helical turn. The same applies to the 2 TG groups in Eq. (7) of the main paper, leading to 1.5 LH turns.

III Extensional Flows

We present extensional flow data at 450 K, and the numbers of right-handed (RH) and left-handed (LH) helices for the three studied temperatures. Figure S1 shows the steady-state nematic order parameter q and the average number of helical turns $\langle N_{\text{turns}} \rangle$ as a function of the Weissenberg number W_i at 450 K. Figure S2 displays $\langle N_{\text{turns}} \rangle$ for RH and LH helices as a function of W_i at 400, 450, and 500 K.

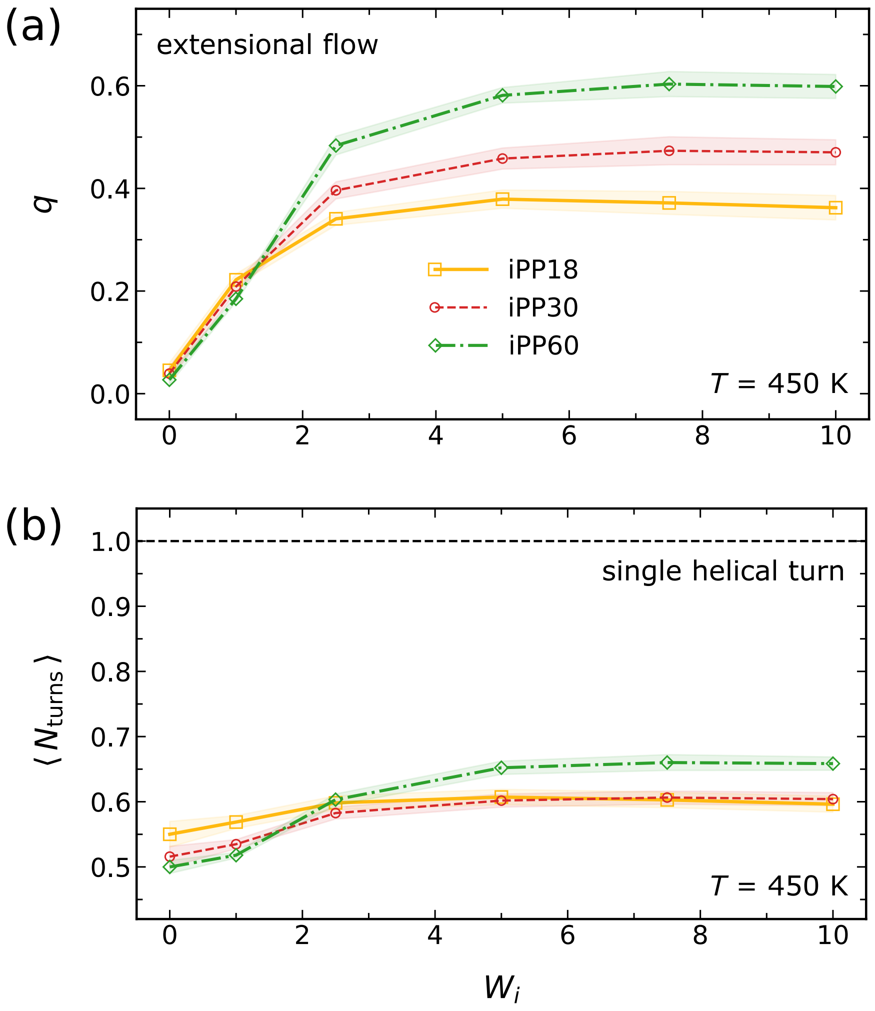

Figure S1: Extensional flow induced steady-state properties at 450 K. (a) Nematic order parameter q as a function of the Weissenberg number W_i. (b) Average number of helical turns $\langle N_{\text{turns}} \rangle$ as a function of W_i. Shades around the curves represent standard deviations.

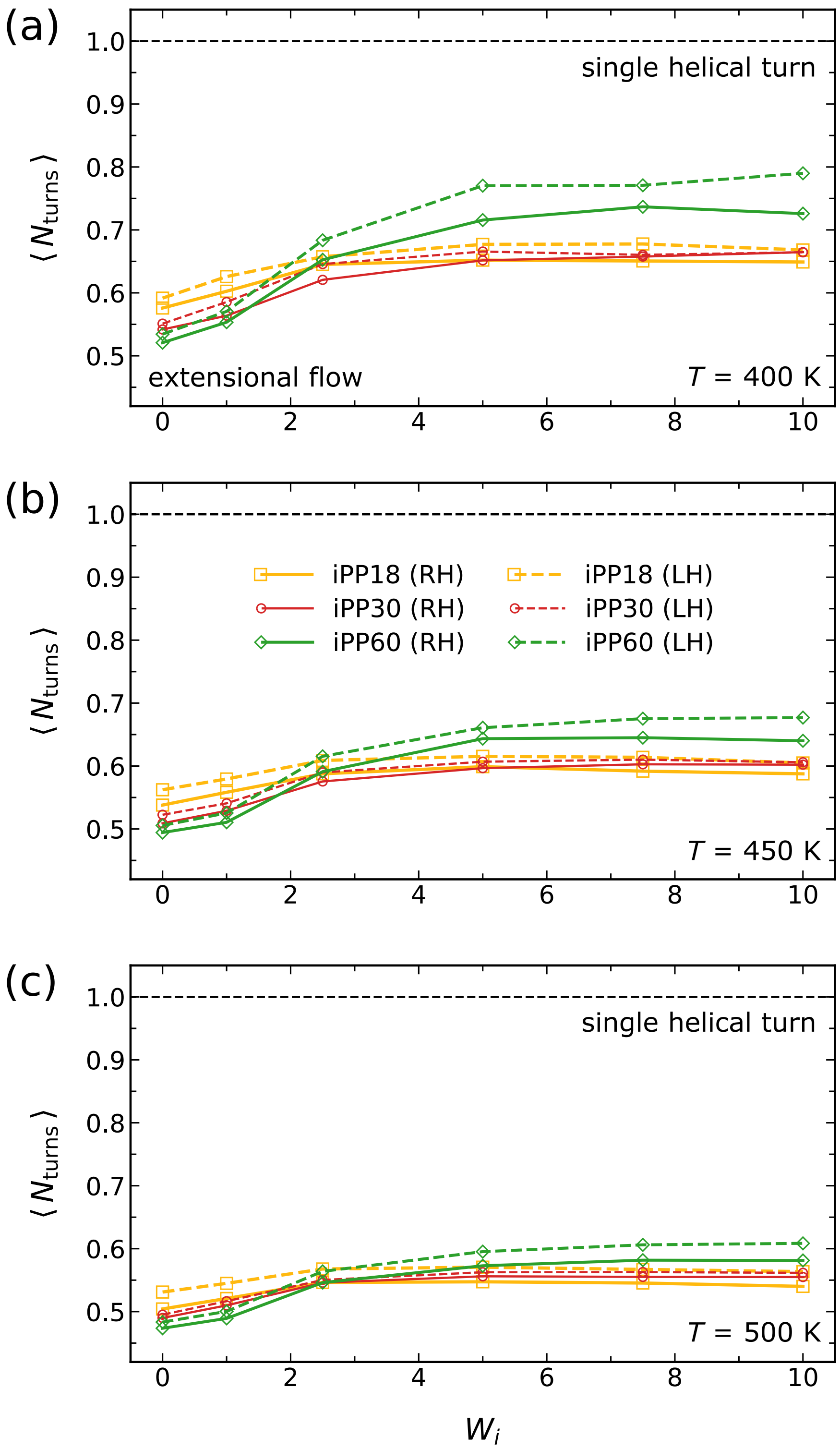

Figure S2: Average number of right-handed (RH) and left-handed (LH) helical turns $\langle N_{\text{turns}} \rangle$ in extensional flows at (a) 400 K, (b) 450 K, and (c) 500 K.

IV Shear Flows

We present shear flow data at 450 K, along with the numbers of right-handed (RH) and left-handed (LH) helices across the three studied temperatures. Figure S1 shows the steady-state nematic order parameter q and the average number of helical turns $\langle N_{\text{turns}} \rangle$ as a function of the Weissenberg number W_i at 450 K. Figure S2 displays $\langle N_{\text{turns}} \rangle$ for RH and LH helices as a function of W_i at 400, 450, and 500 K.

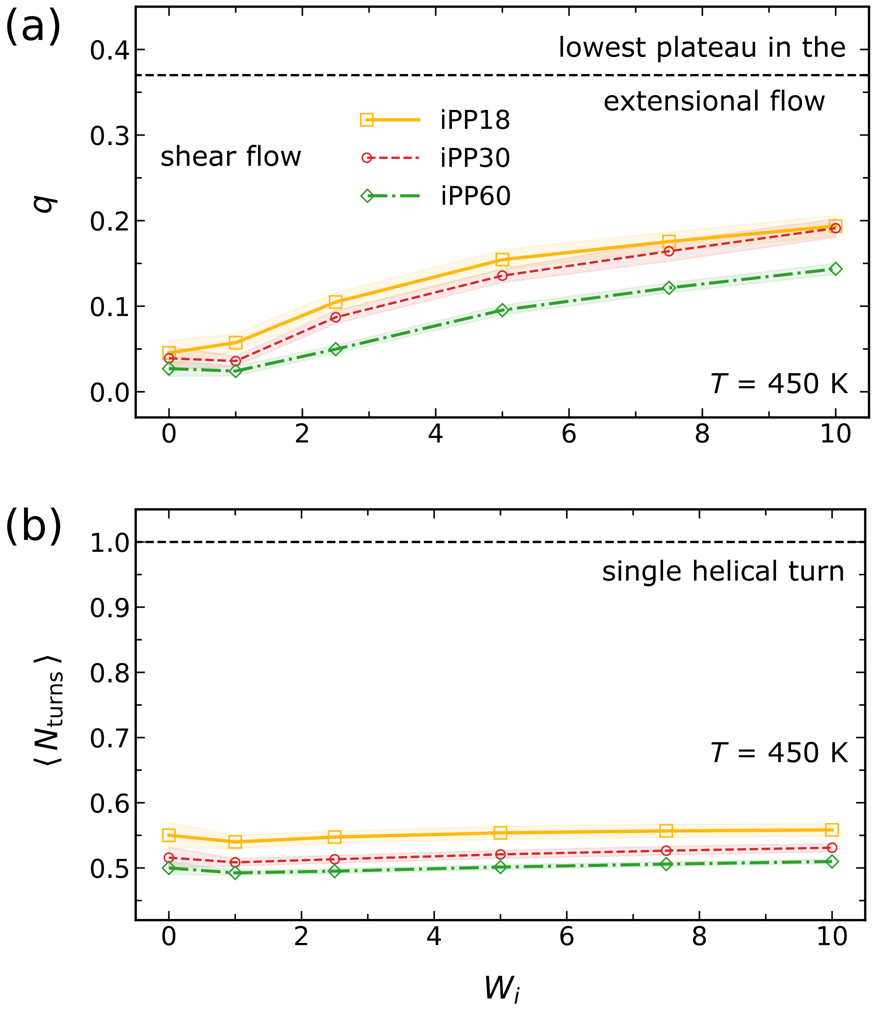

Figure S3: Shear flow induced steady-state properties at 450 K. (a) Nematic order parameter q as a function of the Weissenberg number W_i. (b) Average number of helical turns $\langle N_{\text{turns}} \rangle$ as a function of W_i. Shades around the curves represent standard deviations.

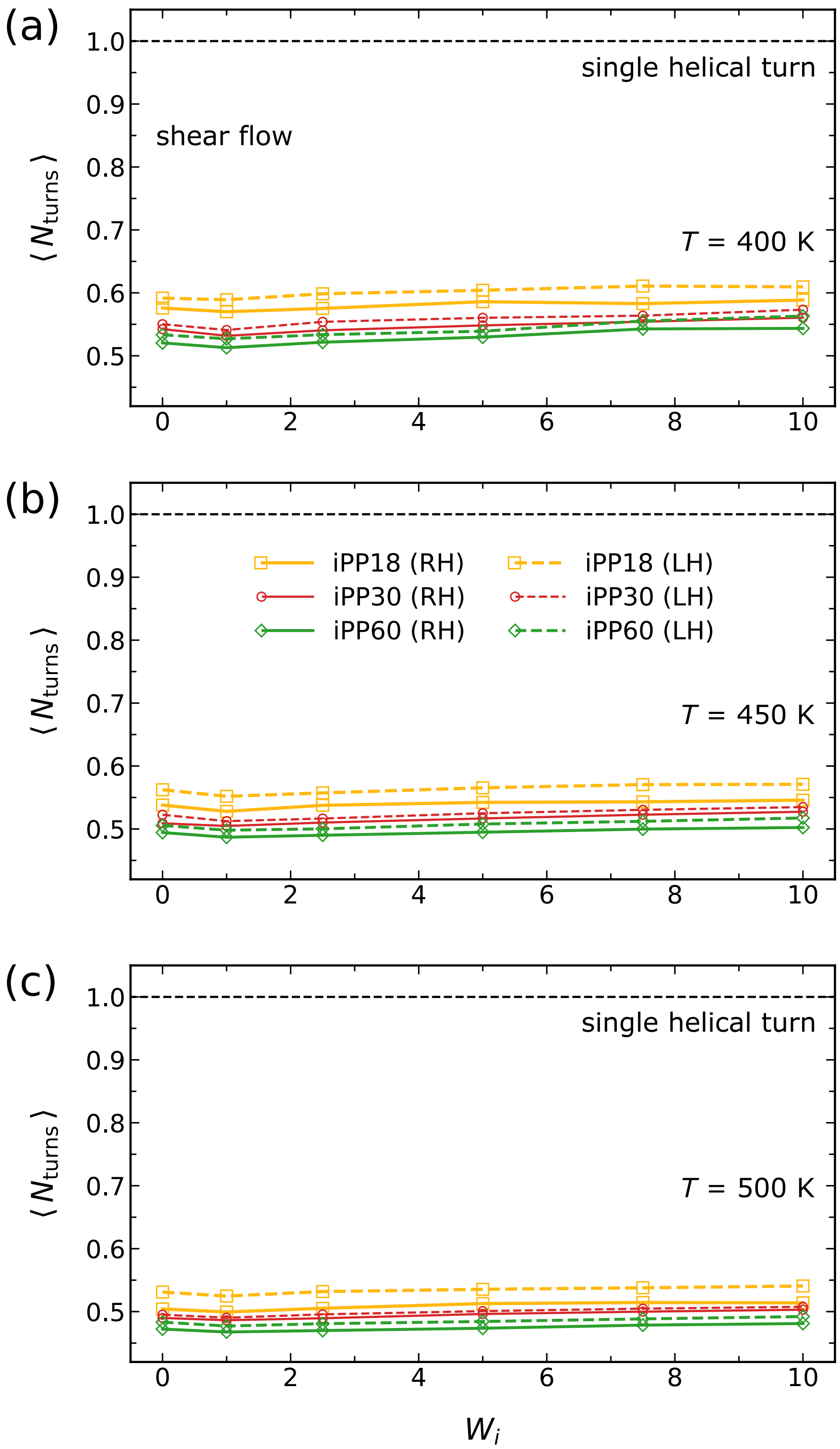

Figure S4: Average number of right-handed (RH) and left-handed (LH) helical turns $\langle N_{\mathrm{turns}} \rangle$ in shear flows at (a) 400 K, (b) 450 K, and (c) 500 K.

V Comparison Between Force Fields

We present extensional flow data for iPP18 at 400 and 500 K using two all-atom force fields: OPLS-AA [4] and COMPASS [5]. We use the same parameters and number of chains specified in Sec. II of the main paper. For COMPASS, we use the publicly available parameters (which do not require a license [6]), and all simulations with this force field are carried out in LAMMPS. The longest Rouse relaxation times τ_R used to compute the Weissenberg number W_i are listed in Table S2, and no significant differences are observed between the two force fields. The nematic order parameter and the average number of helical turns are qualitatively equivalent for both force fields (Fig. S5). The numbers of right-handed (RH) and left-handed (LH) helices are shown in Fig. S6.

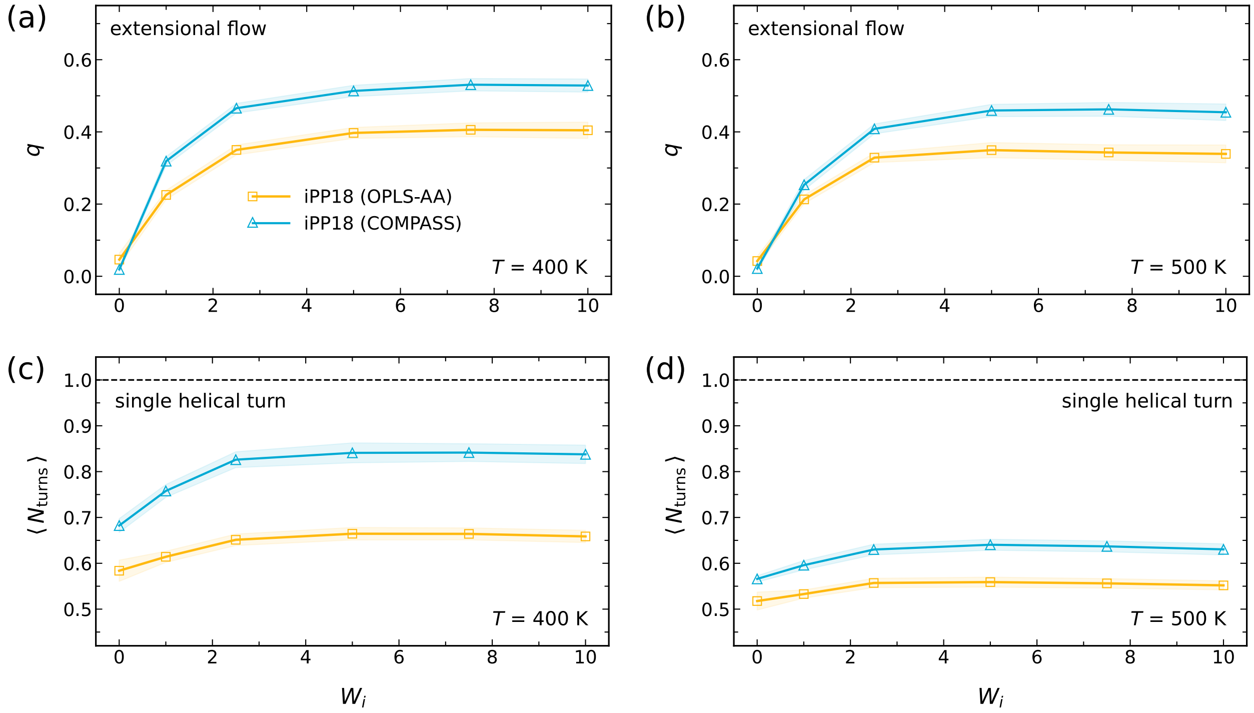

Figure S5: Extensional flow induced steady-state properties of iPP18 for two all-atom force fields. Nematic order parameter q as a function of the Weissenberg number W_i at (a) 400 K and (b) 500 K. Average number of helical turns $\langle N_{\text{turns}} \rangle$ as a function of W_i at (c) 400 K and (d) 500 K. Shades around the curves represent standard deviations.

Table S2: Rouse Relaxation Times τ_R (Eq. (1) in the main paper), in nanoseconds, for iPP18 using two all-atom force fields

	Relaxation Time τ_R (ns)		
iPP18 / Temperature (K)	400	450	500
OPLS-AA [4]	2.1 ± 0.1	0.8 ± 0.02	0.4 ± 0.01
COMPASS [5]	2.5 ± 0.1	0.9 ± 0.02	0.5 ± 0.01

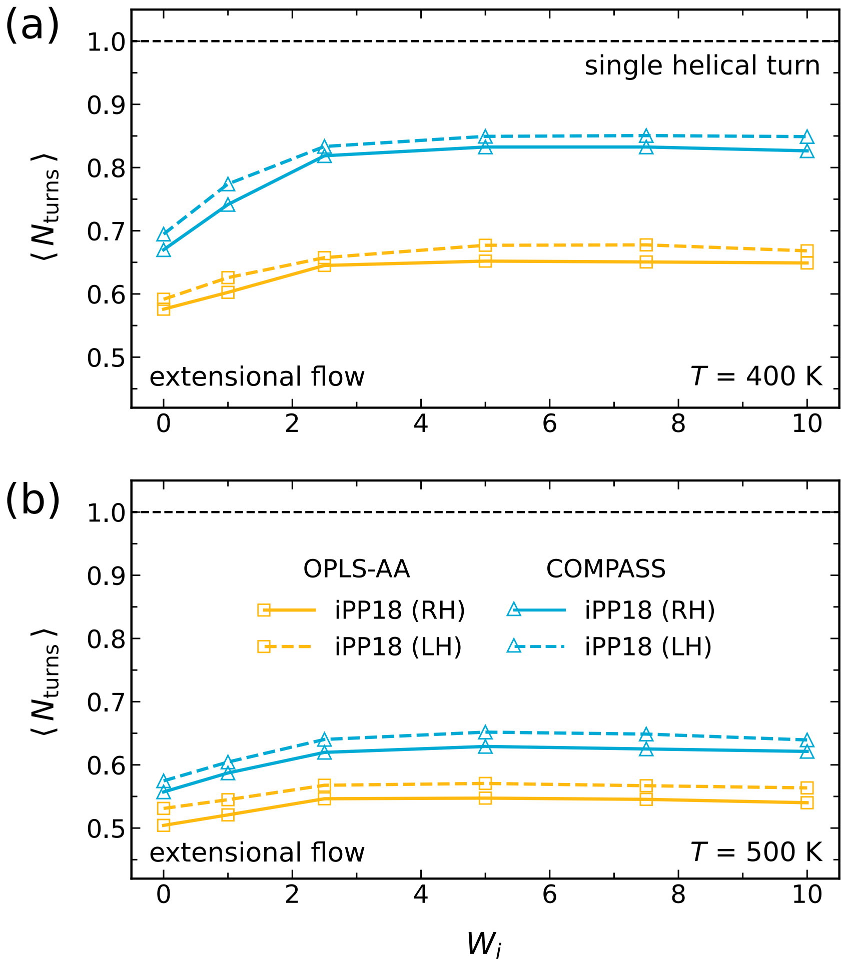

Figure S6: Average number of right-handed (RH) and left-handed (LH) helical turns $\langle N_{\mathrm{turns}} \rangle$ for two all-atom force fields in extensional flows at (a) 400 K and (b) 500 K.

VI Conformational Definitions: Broader Dihedral-Angle Ranges

Xie *et al.* [7] employ broader dihedral ranges to define helical turns, as summarized in Table S3. In Fig. S7, we compare this broader definition with our IUPAC-based definition from Table 2 in the main paper. The broader definition only results in a slight increase in helical turns, indicating that flow simulations do not induce conformational ordering.

Table S3: Broader Dihedral-Angle Ranges Used by Xie *et al.* [7] for Each Rotational Isomeric State (RIS) of iPP

State	Broader Dihedral Range
trans (T)	$(-\pi, -2\pi/3) \bigcup (2\pi/3, \pi]$ [a]
gauche$^-$ (g)	$[-2\pi/3, -\pi/6]$
gauche$^+$ (G)	$[\pi/6, 2\pi/3]$

[a] The symbol $\bigcup$ denotes union of two sets.

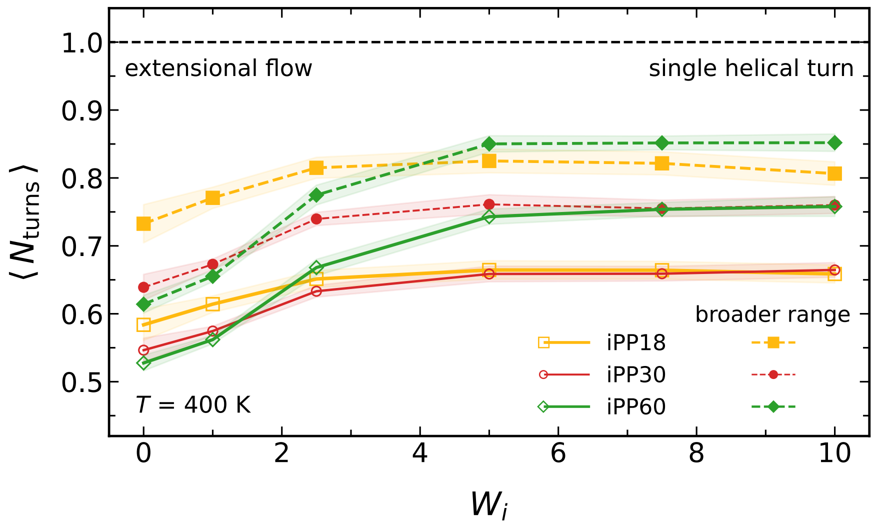

Figure S7: Average number of helical turns $\langle N_{\text{turns}} \rangle$ as a function of W_i using the dihedral ranges from Table 2 of the main paper (unfilled markers) and the broader dihedral ranges from Table S3 above (filled markers). Data are from iPP oligomers under extensional flows at 400 K (see Fig. 4a in the main paper). Shades around the curves represent standard deviations.

VII Quenching iPP Samples

We quench iPP18 samples from extensional flows with $W_i = 10$ at 500 K using two all-atom force fields. No significant differences are observed in the nematic order parameter or in the average number of helical turns between the two force fields (Fig. S8). Once the flow ceases, the aligned iPP18 chains gradually lose their uniaxial alignment. The increase in the number of helical turns—and the subsequent plateaus—arises solely from the reduced thermal fluctuations as the systems are cooled from 500 to 300 K (see Fig. S5 for a comparison between 400 and 500 K).

Figure S9 shows the numbers of right-handed (RH) and left-handed (LH) helices in iPP samples quenched to 300 K.

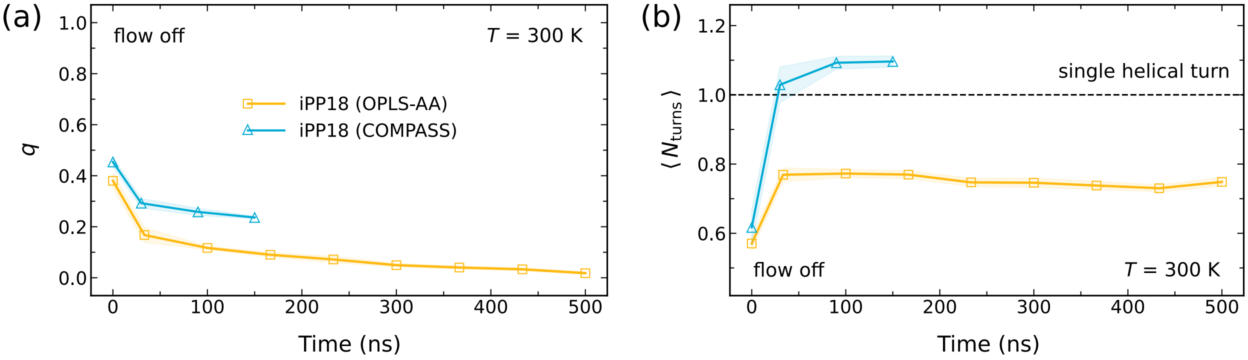

Figure S8: Quenching of iPP18 to 300 K without flow using two all-atom force fields. (a) Nematic order parameter q as a function of time. (b) Average number of helical turns $\langle N_{\text{turns}} \rangle$ as a function of time. Shades around the curves represent standard deviations.

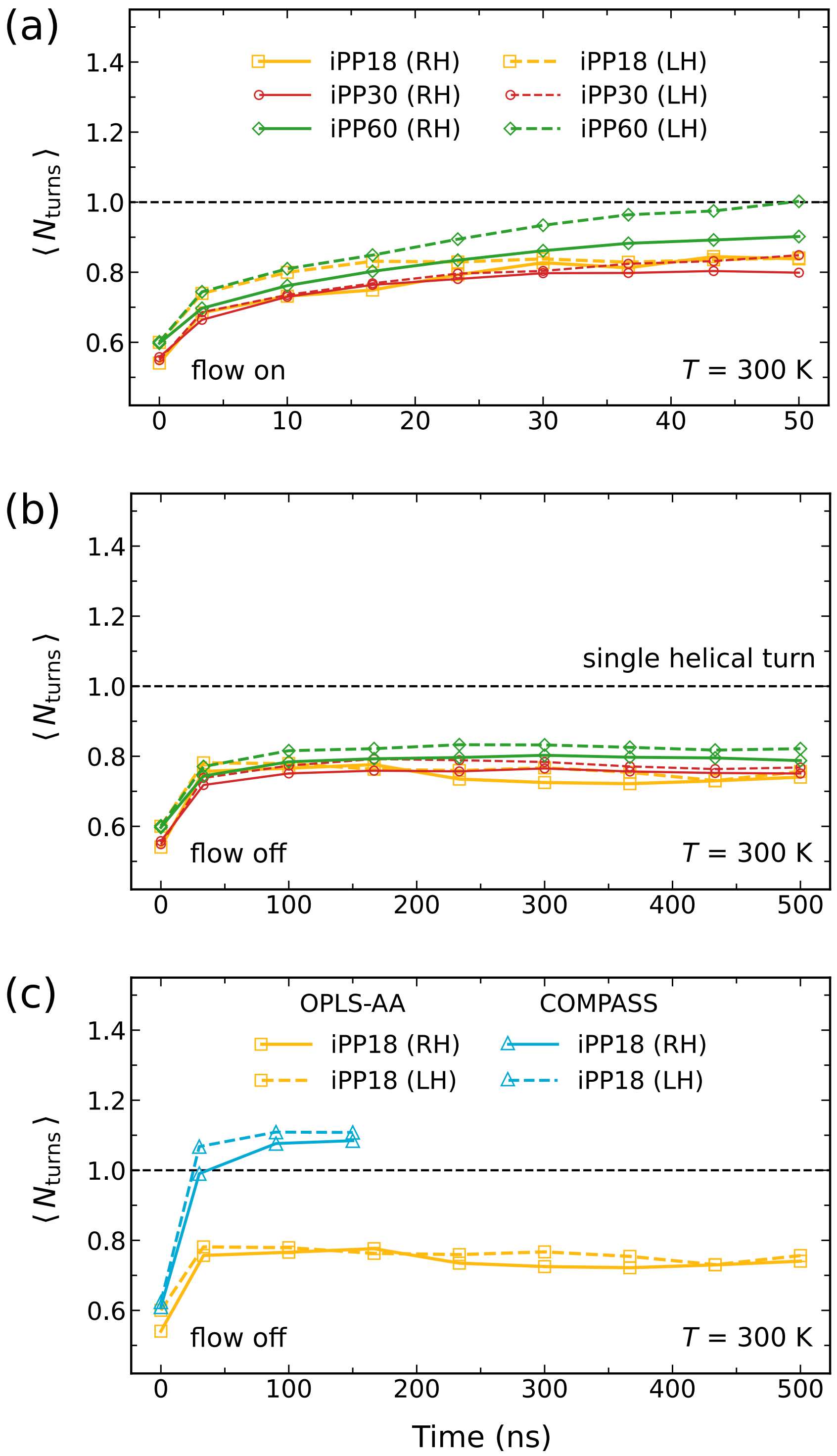

Figure S9: Average number of right-handed (RH) and left-handed (LH) helical turns $\langle N_{\text{turns}} \rangle$ at 300 K: (a) with flow and (b) without flow using OPLS-AA, and (c) without flow for iPP18 using two all-atom force fields. The numbers of RH and LH helices are within one standard deviation of each other and appear statistically indistinguishable.

VIII Dihedral Angle Distribution Analysis

We provide the dihedral angle distributions for the iPP30 samples in Fig. S10. Because iPP30 does not form helical segments under flow (see Fig. 4(b) in the main paper), no significant differences arise between the flow and no-flow distributions. When a dihedral restraint is applied to induce conformational ordering, iPP30 forms helical segments, and the dihedral distribution shifts toward the T, g, and G conformations listed in Table 2 of the main paper. However, based on the definition of helical segments in Sec. II.4 of the main paper, the dihedral distribution alone does not indicate the presence of helical turns.

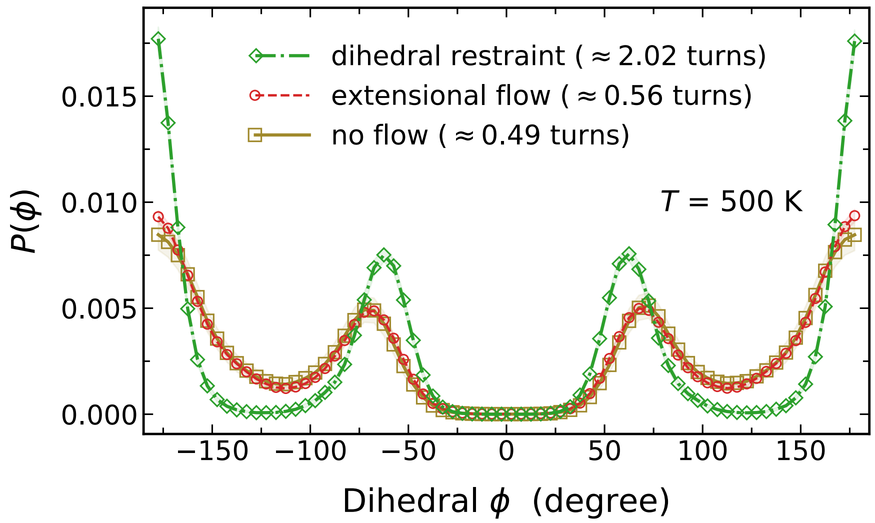

Figure S10: Dihedral angle distribution $P(\phi)$ (probability density) for the iPP30 backbone at 500 K. The samples correspond to simulations with a dihedral restraint using a force constant $\kappa_{\text{dihr}} = 20$ kJ mol^{-1} rad^{-2} (green), extensional flow at $W_i = 10$ (red), and no flow at $W_i = 0$ (yellow) (see Fig. 4(b) in the main paper). The number-averaged helical lengths $\langle N_{\text{turns}} \rangle$ are in parentheses. Shades around the curves represent standard deviations.

IX Orientational and Conformational Contributions to the iPP18 Nucleation Barrier

IX.1 Interfacial Free Energies

To estimate the interfacial free energies between crystalline and molten chains, as defined in Eq. (13) of the main paper, we use the one-dimensional Cahn–Hilliard interfacial functional:[8–10]

$$\gamma[q] = kT \int dz \left[\frac{\zeta}{2} \left(\frac{\partial q}{\partial z} \right)^2 + w\, q^2\, (1-q)^2 \right] , \tag{S2}$$

which is restated here for clarity (see Eq. (14) in the main paper).

To find the function $q(z)$ that minimizes the functional $\gamma[q]$ in Eq. (S2), we set the functional derivative to zero:[11]

$$\frac{\delta \gamma}{\delta q} = 2w\, q\, (q-1)\, (2q-1) - \zeta \frac{\partial^2 q}{\partial z^2} = 0\,, \tag{S3}$$

and define

$$b = \left(\frac{\zeta}{2w} \right)^{1/2} \tag{S4}$$

as the interfacial width. With boundary conditions $q(-\infty) = 0$, $q(\infty) = 1$, and the interface centered at $z = 0$, the solution to Eq. (S3) is:[12]

$$q(z) = \frac{1}{2} \left[1 + \tanh \left(\frac{z}{2b} \right) \right] , \tag{S5}$$

which gives the minimal interfacial free energy upon integrating Eq. (S2) from $-\infty$ to $+\infty$:

$$\gamma = kT \frac{w b}{3}\,. \tag{S6}$$

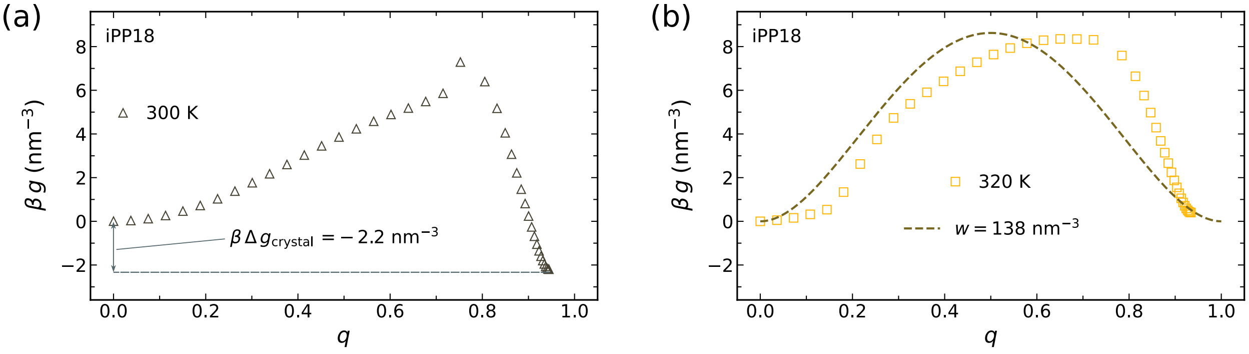

Figure S11: Free energy density βg of iPP18 vs q at (a) 300 K and (b) 320 K. In (a), $\Delta g_{\text{crystal}}$ is estimated from $q = 0$ to $q \to 1$, denoted here as $q = 1$ (see Eq. (19) in the main paper). In (b), we show the fitting used to determine w for the quartic symmetric potential in Eq. (S2). In both panels, βg is computed using the iPP18 free energy data per chain from Fig. 4(b) of Ref. [13].

For iPP18, the minimal interfacial free energies are:

$$\begin{cases} \gamma_{\text{end}} = 5.06\ kT/\text{nm}^2 = 21.0\ \text{mN/m} \quad (\text{at}\ \ T = 300\ \text{K})\ , \\ \gamma_{\text{side}} = 7.82\ kT/\text{nm}^2 = 32.4\ \text{mN/m} \quad (\text{at}\ \ T = 300\ \text{K})\ , \end{cases} \tag{S7}$$

with $w = 138$ nm^{-3}, $b_{\text{end}} = 0.11$ nm, and $b_{\text{side}} = 0.17$ nm. The parameter w is estimated from iPP18 at 320 K (near the melting temperature), where the crystal and melt phases are close to equilibrium and the quartic symmetric potential in Eq. (S2) is better represented. Figure S11 illustrates this estimation, based on the iPP18 free energy data per chain from Fig. 4(b) of Ref. [13]. We convert the data to free energy density using a monomer volume of 0.07 nm^3, estimated from a density of 0.94 g/cm^3 [14] and an iPP molar mass of $MM = 42.08$ g/mol. In the main paper, the same w is used at 300 K as an upper-bound approximation for the interfacial energies γ_{end} and γ_{side} (see Fig. S11 and Eq. (S6)). The interfacial widths in the bundle-end and side directions of the melt-crystal interface are estimated from the iPP crystal structure reported in Ref. [15]. For the bundle-end direction, 3 helical monomers span 0.65 nm, and we take the interfacial width as half of one helical monomer, $b_{\text{end}} = 0.11$ nm. For the side direction, the distance between two iPP chains is 0.67 nm, and the interfacial width is taken as 1/4 of this distance, $b_{\text{side}} = 0.17$ nm. Figure S11 also shows that $\Delta g_{\text{crystal}} = -2.2\ kT/\text{nm}^3$ at 300 K. In the main paper, we note that $\Delta g_{\text{crystal}}$, as used in Eqs. (17) and (18), corresponds to $\Delta g_{\text{crystal}}(0)$ in Eq. (19).

IX.2 Crystallization Entropy Density

We use the RIS method [16–18] to derive the crystallization entropy density per helical turn. We begin with the entropy of crystallization per monomer N:

$$\Delta S_{\text{crystal}} = -\frac{(2N-3)\,k\ln 3}{N}\,, \tag{S8}$$

where $(2N-3)$ is the total number of dihedrals in iPP chains (see Sec. II), k is the Boltzmann constant, and crystallization corresponds to a reduction from 3 possible RIS states (T, G, g) to a single state, contributing $-k\ln 3$ per dihedral. In the limit $N \to \infty$, we obtain $-\Delta S_{\text{crystal}} = 2k\ln 3$, which is consistent with the experimental entropy of fusion for high-molar-mass iPP, (18.9 ± 3.5) J K^{-1} mol^{-1}. [14]

To find the crystallization entropy density per helical turn $\Delta s^{\text{h}}_{\text{crystal}}$ for iPP, we divide $\Delta S_{\text{crystal}}$ in Eq. (S8) by the number of helical turns, $N/3$, and by the monomer volume Ω_{iPP}:

$$\begin{aligned}\Delta s^{\text{h}}_{\text{crystal}} &= -\frac{(2N-3)\,k\ln 3}{N}\,\frac{3}{N}\,\frac{1}{\Omega_{\text{iPP}}}\\ &= -\frac{3\,(2N-3)\,k\ln 3}{\Omega_{\text{iPP}}\,N^2}\,,\end{aligned} \tag{S9}$$

where $\Omega_{\text{iPP}} = 0.07\ \text{nm}^3$, estimated from a density of $0.94\ \text{g/cm}^3$ [14] and the molar mass of an iPP monomer, $MM = 42.08$ g/mol.

IX.3 Estimation of Free Energy Density at 500 K

Using Eq. (S8), we estimate the entropy of crystallization for iPP18 as $\Delta S_{\text{crystal}} = -2.01\ k$. From Fig. S11(b), we find $\Delta g_{\text{crystal}} \approx 0$ at 320 K, which implies $\Delta G_{\text{crystal}}\,(320\ \text{K}) \approx 0$. From this condition, the enthalpy of crystallization is computed as $\Delta H_{\text{crystal}} = -5.36$ kJ/mol. This value agrees well with the negative of the reported enthalpy of fusion for iPP18, -5.2 kJ/mol, in Ref. [19]. Assuming that both $\Delta H_{\text{crystal}}$ and $\Delta S_{\text{crystal}}$ remain approximately constant over the

range 300–500 K, we apply the Gibbs free energy relation

$$\Delta G_{\text{crystal}}(T) = \Delta H_{\text{crystal}} - T\,\Delta S_{\text{crystal}}\,, \tag{S10}$$

to estimate

$$\begin{cases} \Delta G_{\text{crystal}}(500\text{ K}) = 3.01\text{ kJ/mol}\,, \\ \Delta G_{\text{crystal}}(300\text{ K}) = -0.34\text{ kJ/mol}\,, \end{cases} \tag{S11}$$

and the corresponding free energy densities

$$\begin{cases} \Delta g_{\text{crystal}}(500\text{ K}) = 10.34\ kT/\text{nm}^3\,, \\ \Delta g_{\text{crystal}}(300\text{ K}) = -1.95\ kT/\text{nm}^3\,, \end{cases} \tag{S12}$$

where $\Delta g_{\text{crystal}} = \Delta G_{\text{crystal}}/\Omega_{\text{iPP}}$, with $\Omega_{\text{iPP}} = 0.07\text{ nm}^3$ being the iPP monomer volume. This volume is estimated from the density, 0.94 g/cm^3 [14], and the molar mass of an iPP monomer, $MM = 42.08\text{ g/mol}$. Within the approximations, the estimated $\Delta g_{\text{crystal}}(300\text{ K})$ is in good agreement with the value obtained from Fig. S11(a), $\Delta g_{\text{crystal}} = -2.2\ kT/\text{nm}^3$. For the nucleation barrier of iPP18 at 500 K, we use the interfacial free energies from Eq. (S7) at 300 K, assuming they are temperature independent (see Sec. III.4 in the main paper).

References

(1) Duraes, A. D. S.; Gezelter, J. D. Separation of Enantiomers through Local Vorticity: A Screw Model Mechanism. *J. Phys. Chem. B* **2021**, *125*, 11709–11716, DOI: 10.1021/acs.jpcb.1c07127.

(2) Boyd, R. H.; Phillips, P. J. Three Dimensional Architecture: Conformation and Stereochemical Configuration. In *The Science of Polymer Molecules*; Cambridge Solid State Science Series; Cambridge University Press, 1993; Chapter 5, pp 155–187, DOI: 10.1017/CBO9780511600326.

(3) Jones, R. G.; Wilks, E. S.; Metanomski, W. V.; Kahovec, J.; Hess, M.; Stepto, R.; Kitayama, T. *Compendium of Polymer Terminology and Nomenclature: IUPAC Recommendations 2008*; The Royal Society of Chemistry, 2009; Chapter 2, pp 22–43, DOI: 10.1039/9781847559425.

(4) Jorgensen, W. L.; Maxwell, D. S.; Tirado-Rives, J. Development and Testing of the OPLS All-Atom Force Field on Conformational Energetics and Properties of Organic Liquids. *J. Am. Chem. Soc.* **1996**, *118*, 11225–11236, DOI: 10.1021/ja9621760.

(5) Sun, H. COMPASS: An ab Initio Force-Field Optimized for Condensed-Phase ApplicationsOverview with Details on Alkane and Benzene Compounds. *J. Phys. Chem. B* **1998**, *102*, 7338–7364, DOI: 10.1021/jp980939v.

(6) Akkermans, R. L. C.; Spenley, N. A.; Robertson, S. H. COMPASS III: automated fitting workflows and extension to ionic liquids. *Molecular Simulation* **2021**, *47*, 540–551, DOI: 10.1080/08927022.2020.1808215.

(7) Xie, C.; Tang, X.; Yang, J.; Xu, T.; Tian, F.; Li, L. Stretch-Induced Coil–Helix Transition in Isotactic Polypropylene: A Molecular Dynamics Simulation. *Macromolecules* **2018**, *51*, 3994–4002, DOI: 10.1021/acs.macromol.8b00325.

(8) Cahn, J. W.; Hilliard, J. E. Free Energy of a Nonuniform System. I. Interfacial Free Energy. *J. Chem. Phys.* **1958**, *28*, 258–267, DOI: 10.1063/1.1744102.

(9) Cahn, J. W.; Hilliard, J. E. Free Energy of a Nonuniform System. III. Nucleation in a Two-Component Incompressible Fluid. *J. Chem. Phys.* **1959**, *31*, 688–699, DOI: 10.1063/1.1730447.

(10) Wu, H. A Review on the Cahn–Hilliard Equation: Classical Results and Recent Advances in Dynamic Boundary Conditions. *Electron. Res. Arch.* **2022**, *30*, 2788–2832, DOI: 10.3934/era.2022143.

(11) Riley, K. F.; Hobson, M. P.; Bence, S. J. *Mathematical Methods for Physics and Engineering*, 3rd ed.; Cambridge University Press: Cambridge, UK, 2006; Chapter 4, 22, 26, pp 115–150, 775–802, 927–983, DOI: 10.1017/CBO9780511810763.

(12) Wang, S.-L.; Sekerka, R.; Wheeler, A.; Murray, B.; Coriell, S.; Braun, R.; McFadden, G. Thermodynamically-Consistent Phase-Field Models for Solidification. *Phys. D: Nonlinear Phenom.* **1993**, *69*, 189–200, DOI: 10.1016/0167-2789(93)90189-8.

(13) Zhang, W. Thermodynamic Origin of Multistep Polymer Crystallization. *Phys. Rev. Lett.* **2025**, *135*, 028101, DOI: 10.1103/bzpn-qpj5.

(14) Bai, F.; Li, F.; Calhoun, B. H.; Quirk, R. P.; Cheng, S. Z. D. Physical Constants of Poly(propylene). In *Polymer Handbook*, 4th ed.; Brandrup, J., Immergut, E. H., Grulke, E. A., Abe, A., Bloch, D. R., Eds.; John Wiley & Sons, Inc.: New York, USA, 1999; pp V/21–30.

(15) Natta, G.; Corradini, P. Structure and Properties of Isotactic Polypropylene. *Il Nuovo Cimento (1955-1965)* **1960**, *15*, 40–51, DOI: 10.1007/BF02731859.

(16) Strobl, G. Single Chain Conformations. In *The Physics of Polymers: Concepts for Understanding Their Structures and Behavior*; Springer Berlin Heidelberg: Berlin, Heidelberg,

2007; Chapter 2, 5, 8 and 10, pp 15–67, 165–222, 313–356, 415–461, DOI: 10.1007/978-3-540-68411-4.

(17) Rehahn, M.; Mattice, W. L.; Suter, U. W. *Rotational Isomeric State Models in Macromolecular Systems*; Advances in Polymer Science; Springer Berlin Heidelberg: Berlin, Heidelberg, 1997; Vol. 131/132; DOI: 10.1007/BFb0050955.

(18) Tonelli, A. E. Conformational Characteristics of Isotactic Polypropylene. *Macromolecules* **1972**, *5*, 563–566, DOI: 10.1021/ma60029a006.

(19) Chen, Q.; Sirota, E. B.; Zhang, M.; Chung, T. C. M.; Milner, S. T. Free Surfaces Overcome Superheating in Simulated Melting of Isotactic Polypropylene. *Macromolecules* **2015**, *48*, 8885–8896, DOI: 10.1021/acs.macromol.5b02030.
